# Dispersal inference from population genetic variation using a convolutional neural network

**DOI:** 10.1093/genetics/iyad068

**Published:** 2023-04-13

**Authors:** Chris C R Smith, Silas Tittes, Peter L Ralph, Andrew D Kern

**Affiliations:** Institute of Ecology and Evolution, University of Oregon, Eugene, OR 97403, USA; Institute of Ecology and Evolution, University of Oregon, Eugene, OR 97403, USA; Institute of Ecology and Evolution, University of Oregon, Eugene, OR 97403, USA; Institute of Ecology and Evolution, University of Oregon, Eugene, OR 97403, USA

**Keywords:** dispersal, deep learning, space, population genomics, machine learning

## Abstract

The geographic nature of biological dispersal shapes patterns of genetic variation over landscapes, making it possible to infer properties of dispersal from genetic variation data. Here, we present an inference tool that uses geographically distributed genotype data in combination with a convolutional neural network to estimate a critical population parameter: the mean per-generation dispersal distance. Using extensive simulation, we show that our deep learning approach is competitive with or outperforms state-of-the-art methods, particularly at small sample sizes. In addition, we evaluate varying nuisance parameters during training—including population density, demographic history, habitat size, and sampling area—and show that this strategy is effective for estimating dispersal distance when other model parameters are unknown. Whereas competing methods depend on information about local population density or accurate inference of identity-by-descent tracts, our method uses only single-nucleotide-polymorphism data and the spatial scale of sampling as input. Strikingly, and unlike other methods, our method does not use the geographic coordinates of the genotyped individuals. These features make our method, which we call “disperseNN,” a potentially valuable new tool for estimating dispersal distance in nonmodel systems with whole genome data or reduced representation data. We apply disperseNN to 12 different species with publicly available data, yielding reasonable estimates for most species. Importantly, our method estimated consistently larger dispersal distances than mark-recapture calculations in the same species, which may be due to the limited geographic sampling area covered by some mark-recapture studies. Thus genetic tools like ours complement direct methods for improving our understanding of dispersal.

## Introduction

Organisms vary greatly in their capacity to disperse across geographic space. Indeed, the movement of individuals or of gametes across a landscape helps to determine the spatial scale of genetic differentiation and the spread of adaptive variants across natural populations (Broquet and Petit 2009). Consequently, understanding dispersal is relevant for conservation biology (Driscoll *et al.* 2014), studying climate change response and adaptation (Travis *et al.* 2013), managing invasive and disease vector populations (Harris *et al.* 2009; Orsborne *et al.* 2019), phylogeography (Kadereit *et al.* 2005), studying hybrid zones and microbial community ecology (Barton 1979; Evans *et al.* 2017), and for parameterizing models in ecology and evolution (Barton *et al.* 2002). Despite the importance of dispersal, it remains challenging to obtain estimates for dispersal distance in many species.

Some methods infer dispersal distance by directly observing individual movement, using radio-tracking technology, or by tagging and recapturing individuals in the field. However, such measurements can be expensive to obtain and lead to estimates with high uncertainty. Furthermore, they do not always provide a complete picture of *effective* dispersal rate—that is, how far successfully reproducing individuals travel from their birth location (for a review, see Bradburd and Ralph 2019). Effective dispersal is relevant for describing the movement of genetic material across geography, which can be important for understanding population structure, connectivity between populations, evolutionary dynamics of selected alleles, and changes to a species’ range (Slatkin 1987; Peacock 1997).

Another type of method infers effective dispersal distance from genotypes of a single temporal sample, without directly observing movement of individuals. Such inference is possible because population genetics theory predicts how demographic parameters such as the rate of gene flow across the landscape affect the genetic variation of a population (Barton *et al.* 2013). To infer dispersal distance, current population-genetics-based estimators (Rousset 1997; Ringbauer *et al.* 2017) use geographically referenced DNA sequences and can obtain useful estimates of the per-generation dispersal distance, without the need for tracking or recapturing individuals.

Importantly, current population-genetics-based estimators require additional data that can be prohibitively expensive, especially for nonmodel species: for example, an independent estimate of population density (Rousset 1997), or genomic identity-by-descent blocks (Ringbauer *et al.* 2017). Specifically, the seminal method of Rousset (1997) is designed for estimating neighborhood size, $N_{\mathrm{loc}}$, which can be thought of as the number of neighboring individuals or potential mates that are within a few multiples of the dispersal distance (Wright 1946). Wright defined neighborhood size as $N_{\mathrm{loc}}=4\pi D\sigma^{2}$, where *σ* is the dispersal distance and *D* is the population density. Therefore, the accuracy of Rousset’s method depends on having a good a priori estimate of population density. One way to jointly infer dispersal and density works by modeling genomic identity-by-descent tracts (e.g. Barton *et al.* 2013; Baharian *et al.* 2016; Ringbauer *et al.* 2017). The program MAPS (Al-Asadi *et al.* 2019) uses identity-by-descent information to infer heterogeneous dispersal and density across a landscape. Similarly, inferred tree sequences can now be used to infer dispersal rate (Osmond and Coop 2021). Although powerful when applied to high-quality data, the latter methods are limited by the availability of confident identity-by-descent blocks and tree sequences; these data types remain unavailable or difficult to estimate accurately for most species.

Another type of population-genetics-based method estimates *relative* migration rates, for example, EEMS (Petkova *et al.* 2016), FEEMS (Marcus *et al.* 2021), and other landscape genetics tools. Although such methods work well for some applications, such as identifying barriers to dispersal, they do not inform us about the magnitude of dispersal (and so results are not returned with units such as meters per generation). Furthermore, these and related tools model gene flow using an approximate analogy to electrical resistance which can produce misleading results, especially in the presence of biased migration (Lundgren and Ralph 2019). Yet, another class of dispersal estimation uses nonrecombining DNA segments (Neigel *et al.* 1991; Neigel and Avise 1993; Lemey *et al.* 2010), which is valuable for studying phylogeography of viruses, or studying the movement of mitochondrial DNA or sex chromosomes. However, in recombining species, we would ideally leverage more than one locus to infer dispersal rate. In the current paper, we set out to develop a method for estimating dispersal distance that can be applied widely, including in nonmodel species without good assemblies or knowledge of population density.

To do this, we use simulation-based inference via deep learning to estimate dispersal from genotype data. Deep learning is a form of supervised machine learning that builds a complex function between input and output involving successive layers of transformations through a “deep” neural network. An important advantage of this class of methods is their ability to handle many correlated input variables without knowledge of the variables’ joint probability distribution. Like all supervised machine learning methods, deep neural networks can be trained on simulated data, which bypasses the need to obtain empirical data for training (Schrider and Kern 2018). Over the past few years, deep learning has been used in a number of contexts in population genetics: for example, inferring demographic history in *Drosophila* (Sheehan and Song 2016), detection of selective sweeps (Kern and Schrider 2018), detecting adaptive introgression in humans (Gower *et al.* 2021), identifying geographic origin of an individual using their DNA (Battey *et al.* 2020a), and estimating other population genetic parameters like recombination rate (Flagel *et al.* 2019; Adrion *et al.* 2020).

**Fig. 1.**
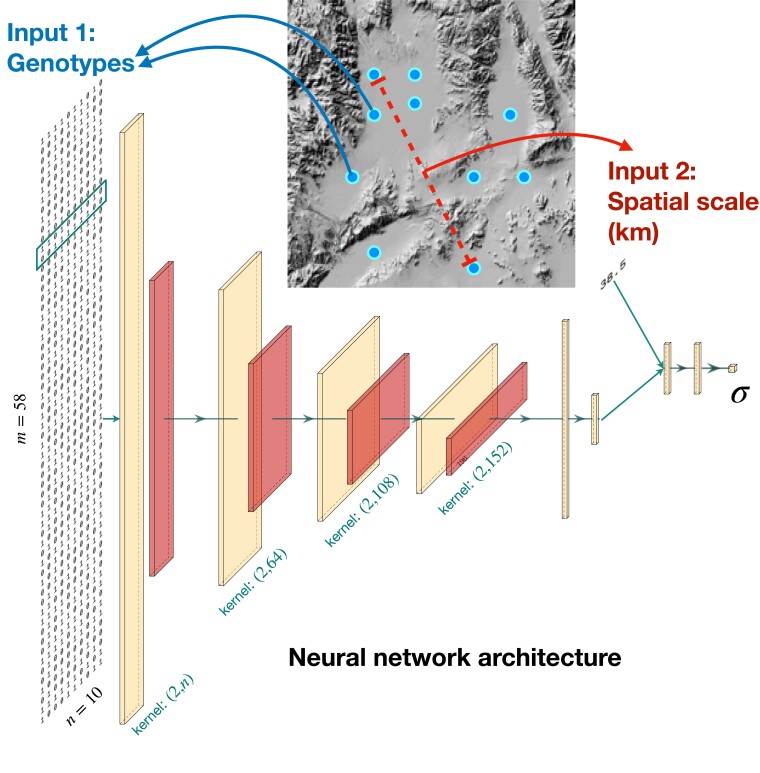
Diagram of the analysis workflow. Points are hypothetical sample locations (n=10) on a geographic map. Rectangular tensors are the output from 1D-convolution layers and average-pooling layers, and the columnar tensors are the outputs from fully connected layers. The number and dimensions of tensors will vary depending on the input dimensions; this example shows a single haplotype for each individual that is 58 (*m*) SNPs long. The box over the genotypes shows the size of the convolution kernel for the first layer. The two input branches are eventually concatenated into a single, intermediate tensor. Neural network schematic generated using PlotNeuralNet (https://github.com/HarisIqbal88/PlotNeuralNet).

We present the first use of deep learning for estimation of spatial population genetic parameters. Our method, called disperseNN (Fig. 1), uses forward in time spatial genetic simulations (Haller and Messer 2019; Battey *et al.* 2020b) to train a deep neural network to infer the mean, per-generation dispersal distance from a single population sample of single nucleotide polymorphism (SNP) genotypes, e.g. whole genome data or RADseq data. We show that disperseNN is more accurate than two existing methods (Rousset 1997; Ringbauer *et al.* 2017), particularly for small to moderate sample sizes, or when identity-by-descent tracts cannot be reliably inferred. After exploring potential shortcomings of our method, we demonstrate its utility on several empirical datasets from a broad range of taxa. The disperseNN software is available from https://github.com/kr-colab/disperseNN, where we have also provided a pretrained model for ease of prediction in new systems.

## Materials and methods

### Simulations

Training datasets were simulated using an individual-based, continuous-space model based on the SLiM model used in Battey *et al.* (2020b). The simulation is initialized with hermaphroditic, diploid individuals distributed randomly on a square habitat. The life cycle of an individual consists of stages for dispersal, reproduction, and mortality. Each offspring disperses from the maternal parent’s location by an independent random displacement in each dimension that is Gaussian distributed with mean zero and standard deviation $\sigma_{f}$. The mate of each individual in each time step is selected randomly, with probability proportional to a Gaussian density with mean zero and standard deviation $\sigma_{m}$, up to a maximum of $3\sigma_{m}$ units in space. The probability of survival of an individual depends on the local population density around the individual, allowing the total population size to fluctuate around an equilibrium. Specifically, the local population density around individual *i* is measured using a Gaussian kernel with standard deviation $\sigma_{c}$ as $n_{i}=\sum_{j}g(d_{ij}/\sigma_{c})$, where g(d) is the standard Gaussian density and $d_{ij}$ is the distance between individuals *i* and *j* and the sum is only over individuals out to a maximum distance of $3\sigma_{c}$. Then, the probability of survival for individual *i* is $p_{i}=\min(0.95,\frac{1}{1+n_{i}/(K(1+L))})$, where *K* and *L* are parameters that are approximately equal to the carrying capacity per unit area and the average lifetime at equilibrium, respectively. In our simulations, L=4 and the number of offspring per mating is Poisson distributed with mean $\frac{1}{L}$. Edge effects are reduced, but not entirely avoided, by decreasing individual fitness proportional to the square root of distance from the habitat edges in units of $\sigma_{c}$. When the proposed location of an offspring would have them disperse outside the bounds of the habitat, the individual is not born, and they are not replaced by another random location. We used a genome length of $10^{8}$ bp and recombination rate $10^{-8}$ crossovers per bp. This model was implemented in SLiM 3.7 (Haller and Messer 2019). Model parameters varied between experiments and the relevant parameter ranges are described in Table 1.

**Table 1.** Parameter distributions used for simulation.

Params.	Description	*σ*	*K*	$N_{1}$	ΔN	Habitat width	Samp. width	Edge
1	Comparing estimators	U(0.245, 3.67)	5	(10,565, 12,463)	Constant	50	1	3
2	Baseline	U(0.245, 3.67)	5	(10,565, 12,463)	Constant	50	1	3
3	Variable density	U(0.245, 3.67)	log-U(0.1, 20.0)	(87, 49,200)	Constant	50	1	3
4	Large density	U(0.245, 3.67)	U(20.0, 40.0)	(45,152, 97,497)	Constant	50	1	3
5	Demographic history	U(0.245, 3.67)	5	(10,565, 12,463)	$\left\{ \begin{array}{c} U(\frac{1}{5},1)\\ U(1,5) \end{array}\right.$	50	1	3
6	Extreme *N* change	U(0.245, 3.67)	5	(10,565, 12,463)	$\left\{ \begin{array}{c} U(\frac{1}{10},\frac{1}{5})\\ U(5,10) \end{array}\right.$	50	1	3
7	Variable habitat size	U(0.245, 3.67)	2	(245, 44,695)	Constant	U(15, 150)	1	*σ*
8	Large habitat size	U(0.245, 3.67)	2	(6030, 176,023)	Constant	U(150, 300)	1	*σ*
9	Variable sampling width	U(0.245, 3.67)	5	(10,565, 12,463)	Constant	50	U(0.2, 0.8)	*σ*
10	Large sampling width	U(0.245, 3.67)	5	(10,565, 12,463)	Constant	50	U(0.8, 1.0)	*σ*
11	Multiple nuisance par.	log-U($1.2\times 10^{-3}$, $1.2\times 10^{2}$)	log-U($10^{-3}$, $10^{4}$)	(163, 301,891)	$\left\{ \begin{array}{c} U(\frac{1}{5},1)\\ U(1,5) \end{array}\right.$	log-U(2, $10^{3}$)	U(0.0, 1.0)	*σ*

The “Params.” column lists the identifier for the parameter set, which is referenced in the main text. “Description” is a brief description of the parameter set. “*σ*” is the distribution of the dispersal parameter. “*K*” is the major determinant of population density. $N_{1}$ is the range of population sizes observed in the final (sampled) generation for each set of simulations. “ΔN” describes the history of population size change: for rows with braces, a random multiplier was chosen from one of two uniform distributions, each with a probability 0.5. The ancestral $N_{e}$ was set to the multiplier × present day *N*. “Habitat width” is for the full habitat. “Samp. width” is the width of the sampling area as a proportion of the full habitat width. “Edge” is a distance from each side of the habitat that was excluded from sampling to avoid edge effects.

In the current paper, we aim to compare disperseNN directly with the methods from Rousset (1997) and Ringbauer *et al.* (2017), who estimate *effective* dispersal. Effective dispersal describes the movement of genetic material over generations and is usually measured as the root mean squared parent-offspring directional displacement, *σ* (i.e. using a model where the displacement in any particular direction has standard deviation *σ*, and the root mean squared Euclidean distance between parent and offspring is $\sqrt{2}\sigma$). In our forward-in-time simulations, *σ* depends on each of the previously mentioned individual-based processes to some extent, which makes it an outcome of the simulation instead of a model parameter. The parameters $\sigma_{f}$ and $\sigma_{m}$ are the primary determinants of *σ*, while the competitive interaction distance, $\sigma_{c}$, has a much smaller effect on *σ*: varying $\sigma_{c}$ tenfold changes *σ* less than 1% (Supplementary Fig. S1). Since *σ* is influenced by more than one parameter, we set $\sigma_{f}=\sigma_{m}=\sigma_{c}$ in the training simulations for disperseNN, which results in a simpler model and a relatively uniform distribution for *σ*.

In the experiments we present in the main text, dispersal history was not recorded during simulations. Therefore, we provide $\sigma_{f}$ as the target to disperseNN during training, and apply a post hoc correction to rescale the predicted values: $\sigma =\sqrt{\frac{3}{2}}\sigma_{f}$. This correction is appropriate because the mean squared directional displacement between mother and child is $\sigma_{f}^{2}$, and between father and child is $\sigma_{f}^{2}+\sigma_{m}^{2}$. So, the mean squared directional displacement between a child and a randomly chosen parent is the average of these two, which if $\sigma_{f}=\sigma_{m}$ is equal to $\frac{3}{2}\sigma_{f}^{2}$. The corrected values are within a few percent of effective *σ* (Supplementary Fig. S2), allowing us to compare the disperseNN output directly with other genetics-based methods. This approach makes the assumption that each of the three types of local interactions occur on the same order, which seems reasonable for some species, but may be inappropriate for others. In a scenario where disperseNN is trained with $\sigma_{f}=\sigma_{m}=\sigma_{c}$, but this assumption is not met in the test data, the *σ* estimate with post hoc correction still works but may be moderately biased (Supplementary Fig. S3).

For tracking effective dispersal (Supplementary Figs. S1–S3) during the simulation, we did the following: let $f_{i}$ be the total number of offspring of individual *i* (acting as either father or mother) and $d_{i}^{2}$ be the average squared displacement along an axis between individual *i* and their parent, averaged over parents and choice of axis (*x* or *y*). We then estimated the effective dispersal distance as $\sqrt{\sum_{i}d_{i}^{2}f_{i}/\sum_{i}f_{i}}$, where the sum is over all individuals alive at any point in the simulation. In experiments where one or more of $\left\{\sigma_{f},\sigma_{m},\sigma_{c}\right\}$ were varied independently of the others, each parameter was drawn from a Uniform(0.2,3).

After the completion of the spatial, forward-in-time SLiM simulation, initial genetic diversity was produced using a coalescent simulation in msprime (Haller *et al.* 2018). This strategy, known as “recapitation,” was necessary to reduce computation time to manageable levels, as the coalescent stage of the simulation is much faster than the spatially explicit portion. The ancestral $N_{e}$ was set to the “present day” census population size for recapitation, as this quantity is more easily observable than effective population size. This portion of the simulation proceeded until all genealogical trees had coalesced. Thus, the complete simulation involves random mating for older generations equivalent to a Wright–Fisher model, with a number of recent generations that are spatially explicit (Table 2). Most of our experiments used 100,000 spatial-SLiM generations. However, to facilitate larger simulations for the multiple nuisance parameters experiment (Parameter Set 11), we ran only 100 generations of spatial SLiM due to computational limitations. This may seem too few, however, since spatial mixing happens over shorter timescales than that of coalescence, then it seems likely that the signal that we are interested in for estimating dispersal would be generated over the recent past. We verified that disperseNN can predict *σ* from full-spatial test data after training on simulations with only 100 spatial generations, although *σ* was moderately underestimated (Supplementary Fig. S4). To simulate population size changes, we recapitated with msprime as before, but included an instantaneous decline or expansion between 100 and 100,000 generations in the past.

**Table 2.** Analysis parameters.

Params.	Description	Sims.	Training	Spatial gen.	*n*	SNPs	Phased
1	Comparing estimators	1,000	50,000	100,000	10 and 100	$2.5\times 10^{5},5\times 10^{5}$	Y
2	Baseline	1,000	50,000	100,000	100	5,000	Y
3	Variable density	1,000	50,000	100,000	100	5,000	Y
4	Large density	1,000	50,000	100,000	100	5,000	Y
5	Demographic history	1,000	50,000	1,000	100	5,000	Y
6	Extreme *N* change	1,000	50,000	1,000	100	5,000	Y
7	Variable habitat size	1,000	50,000	100,000	50	5,000	Y
8	Large habitat size	1,000	50,000	100,000	50	5,000	Y
9	Variable sampling width	1,000	50,000	100,000	100	5,000	Y
10	Large sampling width	1,000	50,000	100,000	100	5,000	Y
11	Multiple nuisance par.	2,300	100,000	100	U-int(10,100)	5,000	N

The “Params.” column lists the identifier for the parameter set, which is referenced in the main text. “Description” is a brief description of the parameter set. “Sims.” is the number of true replicates, i.e. SLiM simulations, represented in training. “Training” is the size of the total training set after drawing multiple samples from each simulation. “Spatial gen.” is the number of spatial generations simulated in SLiM. “*n*” is the sample size. “SNPs” is the number of SNPs used in training. “Phased” describes whether the data were phased or not for training. See Table 1 for corresponding distributions for parameters of the simulation model.

**Fig. 2.**
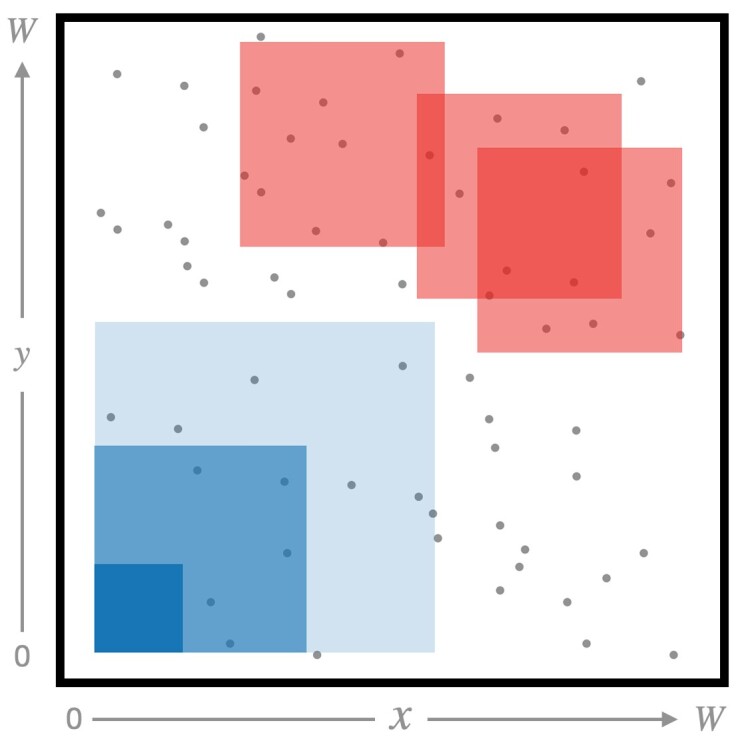
Cartoon showing different sampling strategies. The large box represents the full simulated habitat. For some experiments, we both (i) varied the width of the square sampling window—see the boxes with different sizes—, and (ii) assigned a uniform-random position for the sampling window—see the boxes with different positions.

Individuals were sampled randomly from within the sampling window. When the specified size of the spatial sampling window was smaller than the full habitat, the position of the sampling window was chosen randomly, with *x* and *y* each distributed uniformly (Fig. 2), excluding edges. The amount of edge cropped was either set to (i) *σ* for each simulation, or (ii) the maximum of the simulated *σ* range for the whole training set, depending on which simulation parameters were free to vary; the latter was necessary to avoid information leakage during training. For some analyses, multiple, partially overlapping samples were drawn from the same simulation to save computation time; these cases are noted in Table 2. This strategy allows for large training sets to be generated from a smaller number of starting simulations.

To obtain genetic data at *m* varying sites, neutral mutations were superimposed on the tree sequences using msprime v1.0 (Baumdicker *et al.* 2022) (for values of *m* see the “SNPs” column in Table 2). This mutate-afterwords approach is efficient because we only add mutations that affect the sampled individuals (Kelleher and Lohse 2020). For efficiency, we used an iterative approach for adding mutations because our analyses used a fixed, and usually small, number of mutations. Specifically, we started by simulating mutations with a very small mutation rate, $10^{-15}$. If we did not yet have *m* SNPs, we increased the mutation rate by 10x, and added additional mutations with the updated mutation rate. This was iterated until at least *m* mutations had been obtained. Finally, a uniform sample of *m* biallelic SNPs were sampled to represent the genotype matrix input to disperseNN. The result of this procedure is that the genotype matrix for each simulated dataset contains the same number of SNPs, *m*, regardless of the actual number of variable sites in the sampled individuals, and irrespective of mutation rate. Thanks to the Poisson nature of neutral mutations, this procedure is equivalent to having simulated with a higher mutation rate and randomly selected *m* variable sites.

The input for disperseNN consists of two things: the width of the spatial sampling area, and a genotype matrix, having one row for each SNP and one or two columns per individual depending on the phasing designation. If phased, the genotype matrix contained two columns per individual, randomly ordered, with 0s and 1s encoding minor and major alleles, respectively. If unphased, the genotype matrix contained one column per individual with genotypes encoded as 0s, 1s, and 2s, representing the count of the minor allele. In order to facilitate various sample sizes in real applications, our pretrained model used a random sample size during training, 10≤n≤100, with zero padding out to 100 columns. To obtain the second input, we used the furthest distance between pairs of samples as the sampling width. The training targets are the true values of log(σ). Thus, the output from the network is in log space (disperseNN exponentiates the result before writing the predictions).

In generating training data for the pretrained network, we sought to explore a large parameter range: each parameter varied over several orders of magnitude (Parameter Set 11). However, swaths of parameter space described by the ranges in Table 1 were not represented in the training data, due to the following logistical hurdles. First, simulations, where the population died, were not included in the training set. The excluded simulations had small carrying capacity and small habitat size, or small habitat size and large *σ*, for example. Next, some simulations could not be run due to computational constraints: maximum RAM of 175 gigabytes and two-week wall time on our computing cluster. For example, combinations of large carrying capacity and large habitat size were not simulated. As a result, only 12% of attempted simulations were included in training, and for each parameter the *realized* distribution—representing successful simulations—differed from the distribution from which the model settings were drawn (Supplementary Fig. S5), which had been uniform in log space.

### Network architecture and training

Tensorflow (Abadi *et al.* 2016) and Keras (https://github.com/keras-team/keras) libraries were used to develop disperseNN. The first input tensor, the genotype matrix, goes through successive convolution and pooling layers, a strategy that is characteristic of convolutional neural networks (CNNs) (Fig. 1). We adjusted the number of convolution and pooling layers based on the size of the genotype matrix: the number of convolution layers assigned was equal to floor(log_10_(number of SNPs))−1. The filter size of successive convolution layers was 64 for the first layer, and 44 larger for each successive layer. The convolution layers are one-dimensional, such that the convolution kernel spans all individuals (columns) and two SNPs (rows), with a stride size equal to one. Average pooling layers were also one-dimensional, spanning all individuals and 10 SNPs. After the convolutional portion of the network, the intermediate tensor was flattened and put through three fully connected layers each with 128 units and rectified linear unit (ReLU) activation. A second input branch was used for the sampling area. This input tensor with size =1 was concatenated with the preceding branch, then subjected to a 128-unit dense layer with ReLu. Finally, a dense layer with linear activation was applied which outputs a single value, the estimate for *σ*.

During training, we held out 20% of the training set for computing a validation-loss between epochs. We used a batch size of 40, mean squared error loss, and the Adam optimizer. The learning rate was initialized as $10^{-3}$. The “patience” hyperparameter determines both the length of training, and learning rate adaptations during training: after a number of epochs equal to patience/10 without improvement in validation loss the learning rate is halved, and training proceeds until a number of epochs equal to patience pass without improvement in validation loss. Patience was set to 100 for all training runs excluding the pretrained model. For the pretrained model, we explored a grid of different hyperparameter settings: patience values of 10, 20, 30, 40, and 50; initial learning rates of $10^{-4}$, $10^{-3}$, and $10^{-2}$; and dropout proportions of 0, 0.1, 0.2, and 0.3. We landed on settings that consistently gave the lowest MRAE: patience =10, initial learning rate of $10^{-3}$, and 0 dropout.

The architecture we present does not use spatial information, except for a scalar for the width of the sampling area. Although we focus on the nonspatial architecture, an extension to our model might incorporate the relative spatial distances between samples. For the goal of conveying spatial information, we have explored basic architectures that did not work well, at least with uniform sampling (see Appendix); however, the spatially explicit architectures may help differentiate spatial sampling strategies (e.g. point sampling, transect sampling, etc.), which we have not yet explored. Many alternative architectures are conceivable and may be analyzed in future studies. For example, the spatial connectedness between individuals might be shown to the network by sorting the genotypes by their geographic coordinates.

### Comparison with other estimators

The method from Rousset (1997) uses the observation that under certain assumptions $b=\frac{1}{4\pi D\sigma^{2}}$, where *D* is density and *b* is the slope of the least squares fit of $a_{r}/(1-a_{r})$ to log geographic distance. Here, $a_{r}$ is a measure of genetic differentiation, analogous to $F_{st}$, that can be estimated for a pair of individuals, P, as ${\hat{a}}^{*}=\frac{(2SS_{b(P)}-SS_{W(P)})P}{2\sum_{k=1}^{P}SS_{W(k)}}$ . In this equation, $SS_{b(P)}$ is the sum of squared differences between the two individuals’ genotypes, $SS_{W(P)}$ is the sum of squared differences between genomes within the individuals, *P* is the total number of pairs of individuals in the sample, and $\sum_{k}^{P}SS_{W(k)}$ are within individual differences summed over the *P* different pairs of individuals. We applied Rousset’s method to the same genotypes and sample locations as for disperseNN. The value for *D* used with this method was calculated using the following procedure. First, $N_{e}$ was calculated using the “inbreeding effective size” from Waples (2002):


(1) $$N_{eI}=\frac{N\bar{k}-2}{(\bar{k}-1)+V_{k}/\bar{k}}$$

where $\bar{k}$ is the mean number of offspring per individual and $V_{k}$ is the variance in offspring number among individuals. To deal with overlapping generations, we calculated $N_{eI}$ each simulation cycle using census *N* from the current cycle along with the lifetime offspring count, *k*, for dying individuals. The per generation mean $N_{eI}$ was calculated at the end of the simulation. With an estimate for $N_{e}$ in hand, we calculate density as $D=N_{e}/(W-2\sigma {)}^{2}$, where the 2σ is to exclude edges which have reduced density.

A second comparison was made with IBD-Analysis (Ringbauer *et al.* 2017). The authors used the distribution of identity-by-descent tract lengths shared between individuals to estimate *σ*. They derived analytical formulas describing the distribution of identity-by-descent tract lengths in continuous space and provided an inference scheme that uses maximum likelihood to fit these formulas. For our comparison, we applied two different pipelines with IBD-Analysis. First, we extracted the true identity-by-descent tracts directly from the tree sequences output from SLiM. Specifically, for each pair of individuals, for each combination of chromosomes between the individuals, we simplified the tree sequence to represent only the recombination history between the two chromosomes, and extracted segments that were inherited from a common ancestor without recombination. These were the identity-by-descent tracts used as input for the IBD-Analysis program, which was obtained from https://git.ist.ac.at/harald.ringbauer/IBD-Analysis. Separately, we inferred identity-by-descent tracts in the simulated data using an empirical tool, Refined IBD (Browning and Browning 2013), and used the inferred identity-by-descent blocks as input IBD-Analysis. We used the following IBD-Analysis settings: genome length of 1e8 bp, the minimum block length of 1 cM (default). We used the following Refined IBD settings: minimum block length of 1 cM (default=1.5 cM).

### Empirical data

To demonstrate the utility of disperseNN, we applied it to preexisting publicly available datasets that have the following criteria: spatially distributed genetic data, latitude and longitude metadata available, ten or more sampling locations, sampling area less than 1,000 km, at least 5,000 biallelic SNPs, and a ready-to-plug-in SNP table that had been processed and filtered by the original authors. For some datasets with overall sampling width more than 1000 km, we were able to subset to a smaller cluster of sample locations (see details specific to each dataset below). When multiple individuals were sampled from the same location we chose one random individual from each location, in order to better match the sampling scheme used in generating training data. SNP tables were converted to genotype matrices after minimal processing: we removed indels and sites with only one, or more than two, alleles represented in the sampled subset. We required all sampled individuals to be genotyped to retain a SNP, except when we note otherwise—see details specific to each dataset below.

Mosquito data were downloaded following instructions from https://malariagen.github.io/vector-data/ag3/download.html. We used a dense cluster of sampling localities in Cameroon that had been identified as *Anopheles gambiae*. Individual VCFs were merged using bcftools (v1.14). Chromosomes 3L and 3R were analyzed; 2L and 2R were excluded due to previously reported large inversions (Lobo *et al.* 2010; Riehle *et al.* 2017).

*Arabidopsis* data were downloaded from https://1001genomes.org/data/GMI-MPI/releases/v3.1/ as a single VCF. Two conspicuous geographic clusters were chosen from Sweden and Spain to minimize the geographic sampling area. All five chromosomes were analyzed.

Sunflower data were downloaded following instructions from https://rieseberglab.github.io/ubc-sunflower-genome/documentation/. Geographic clusters of sampling localities were identified in Texas (*Helianthus argophyllus*) and on the border of Kansas and Oklahoma (*H. petiolaris*). Individual VCFs were merged into multi-sample VCFs for each of the two species. Chromosomes 1–17 were analyzed, excluding a number of unplaced scaffolds.

VCFs for oyster (*Crassostrea virginica*; Bernatchez *et al.* 2019), bumble bee (*Bombus*; Jackson *et al.* 2018), Atlantic halibut (*Hippoglossus hippoglossus*; Kess *et al.* 2021), white-footed mouse (*Peromyscus leucopus*; Munshi-South *et al.* 2016), Réunion grey white-eye (*Zosterops borbonicus*) and Réunion olive white-eye (*Zosterops olivaceus*; Gabrielli *et al.* 2020), and wolf (*Canis lupus*; Schweizer *et al.* 2016) were downloaded directly from The Dryad Digital Repository. Clusters of sample locations were chosen in each dataset to maximize sampling density. In the datasets from *Bombus vosnesenskii*, *Peromyscus leucopus*, *Zosterops borbonicus*, and *Zosterops olivaceus*, we allowed as few as 85%, 60%, 90%, and 90% of individuals to be genotyped to retain a SNP, respectively, and missing genotypes were filled in with the major variant.

To calculate the width of the sampling window for empirical data, we calculated the geodesic distance between each pair of individuals using the package geopy with the WGS84 ellipsoid. This distance represents the shortest path on the surface of the Earth between points. The longest distance between pairs of sample locations was used as the sampling width, which we provided in kilometers to disperseNN.

## Results

### Dispersal estimation using a deep neural network

We use a CNN trained on simulated data to infer parent-offspring distance (Rousset 1997; Ringbauer *et al.* 2017) (Fig. 1). Concretely, we aim to infer *σ*, defined here as the root-mean-square displacement along a given axis between a randomly chosen child and one of their parents chosen at random (and, we assume directional invariance, so this does not depend on the axis chosen). The CNN takes two pieces of data as input: (1) a genotype matrix, and (2) the distance (e.g. in km) between the two furthest geographic samples. The genotype matrix is put through the network’s convolution layers, while the sampling width is used downstream to convey the physical scale of sampling. The output from the CNN is a single value, an estimate of *σ*. Our software package, disperseNN, has several inference-related functionalities: training the CNN on simulated data, predicting *σ* using simulated or empirical data, and preprocessing steps for empirical data. In addition, disperseNN includes a pretrained network that can be used to estimate dispersal without additional training; although the quality of the estimate will depend on how well the data fit the conditions used in training this network; see below for discussion.

It is well-known that neighborhood size and dispersal rate not only create genetic patterns of isolation-by-distance, but also influence other facets of population genetic variation. Dispersal affects the site frequency spectrum and other standard summary statistics, such as *θ*, heterozygosity, F_*is*_, Tajima’s D, the variance in D_*xy*_, or nIBS (see, for example, Battey *et al.* 2020b). Therefore, a natural strategy for estimating *σ* might involve the aforementioned summary statistics, or, as researchers have done for other tasks (e.g. Flagel *et al.* 2019), use machine learning to extract relevant features from the genotypes themselves in an automated fashion; this is what we seek to do with disperseNN.

The convolutional design we use in disperseNN is intended to be flexible with respect to input data, as we imagine the use case to be everything from RADseq with minimal, short-range linkage information to whole genome sequence data, where linkage information would be fully preserved. The initial convolutional kernel spans the genotypes of all individuals at once (i.e. a one-dimensional convolution layer), in a manner that allows the network to glean population-level information at each site, and strides across SNPs two at a time, to make use of correlation patterns when linked SNPs are available. Likewise, we use successive layers of data compression, through convolution and pooling, to coerce disperseNN to look at the genotypes at different scales and hopefully to learn the extent of linkage disequilibrium. Counterintuitively, our approach deviates from other dispersal estimators (Rousset 1997; Ringbauer *et al.* 2017) because it does not directly utilize the spatial coordinates of sampled individuals, except for calculating the width of the sampling area.

The training data for disperseNN are generated using a continuous-space SLiM model adapted from Battey *et al.* (2020b). Training with disperseNN consists of: deciding on training distributions for *σ* and other parameters of the spatial model, simulating training data, and handing the simulation output and targets (true values of *σ*) to disperseNN for training the CNN. The analysis pipeline for predicting on simulated data is similar to the training pipeline, while predicting on empirical data involves basic preprocessing of the input data before using disperseNN to estimate *σ*. Below, we present findings from several experiments using disperseNN, each with its own set of parameters for simulation and training. We describe each experiment briefly in the Results section, and reference different sets of parameters that correspond to each experiment, e.g. “Parameter Set 1,” “Parameter Set 2,” etc. Full details about the different parameter sets are in the Materials and Methods section.

### Comparison with existing methods

We evaluated the accuracy of our method on simulated datasets with a range of *σ* values, using the relative absolute error (RAE) to measure prediction accuracy for each estimate:


(2) $$\mathrm{RAE}=|\frac{\mathrm{estimated}\;\sigma -\mathrm{true}\;\sigma }{\mathrm{true}\;\sigma }|$$

**Fig. 3.**
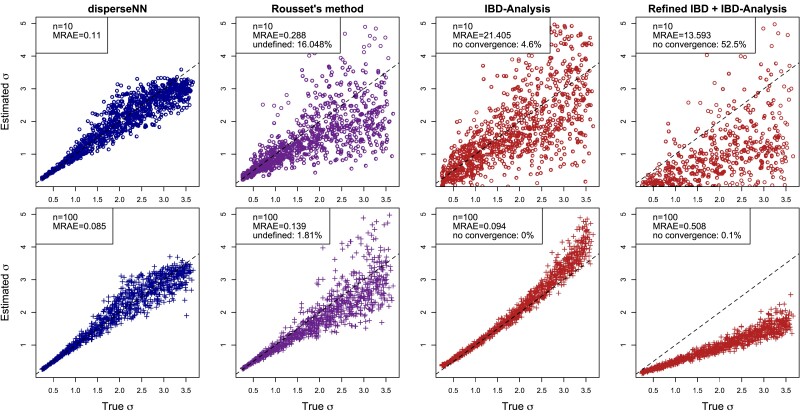
Comparison with existing methods (Parameter Set 1). Here, disperseNN is compared with Rousset’s method and the method of Ringbauer *et al.* (2017) with both true identity by descent tracts (“IBD-Analysis”) and tracts inferred by Refined IBD (“Refined IBD  +  IBD-Analysis”). Two different numbers of sampled individuals are shown: n=10 (top row) and n=100 (bottom row). The numbers of SNPs used with each sample size were $2.5\times 10^{5}$ and $5\times 10^{5}$, respectively. The dashed lines are y=x. Estimates greater than 5 are excluded from plots but are included in the MRAE calculation. Methods other than disperseNN sometimes produced undefined output; these data do not contribute to the MRAE. The MRAE for IBD-Analysis is greater than Refined IBD  +  IBD-Analysis with n=10 due to outlier points that inflated the MRAE using the former method and caused undefined output with the latter method.

For comparing accuracy between training runs or between methods, we calculate the mean relative absolute error (MRAE) averaged across all test datasets. We found disperseNN estimates dispersal rate more accurately than previous genetics-based methods (Fig. 3; Parameter Set 1). At small sample sizes (n=10), disperseNN was dramatically more accurate than the Rousset (1997) method, the program from Ringbauer *et al.* (2017) called IBD-Analysis run with true identity-by-descent blocks (available from recorded genealogies), and IBD-Analysis used with empirically estimated identity-by-descent blocks (MRAE values of 0.11, 0.33, 21.41, and 13.6, respectively). Furthermore, methods other than disperseNN produced undefined output or convergence errors for 16.4%, 4.6%, and 52.5% of test datasets, respectively. For Rousset’s method, this is due to a negative slope in the least squares fit of genetic distance versus geographic distance, which happens more frequently with a small sample size and larger *σ*. In addition, Rousset’s method and IBD-Analysis occasionally produced extreme overestimates for larger *σ* values.

With a larger sample size (n=100), the accuracy of each method improved to varying degrees, with disperseNN and IBD-Analysis with true identity-by-descent information performing similarly (MRAE=0.085, 0.23, and 0.094, respectively). As can be seen in Fig. 3, IBD-Analysis is extremely accurate with large sample sizes and true identity-by-descent tract information, but had substantially worse performance when tracts must be inferred from genotype data (as would happen in practice). However, despite being underestimated by a constant factor when using empirically inferred tracts, IBD-Analysis still seemed to capture a signal of dispersal rate. IBD-Analysis with true tracts overestimated *σ* towards the larger end of examined range (IBD-Analysis’s bias may be due to limits related to inferring large *σ* relative to the habitat size, or alternatively due to sampling uniformly at random instead of regularly spaced in a grid as in Ringbauer *et al.* 2017.) In this comparison disperseNN has the advantage of being trained using the true distribution of *σ*; it expects a certain range of *σ* values, which helps it avoid extreme outliers during prediction.

Larger numbers of SNPs improved the performance of disperseNN relative to the other methods, although with diminishing returns. See Supplementary Figs. S6 and S7 for results with fewer SNPs. As the number of SNPs decreased, the Rousset method retained good accuracy, better than disperseNN for small *σ* values with n=100 samples (although not n=10). For every input size, larger values of *σ* were inferred with correspondingly larger errors (Fig. 3), however, *relative* error was nearly constant across the range of true *σ* for disperseNN (Supplementary Fig. S8). In addition, disperseNN slightly underestimated *σ*, and Rousset’s method had larger relative error, when the true value approached the maximum of the examined range. This likely occurs because *σ* can only be so large before approximate random mating is reached; it is difficult to distinguish a large *σ* from a larger-*σ* if both populations have approximately random mating.

### The effect of model misspecification, and how to fix it

A common concern with supervised machine learning methods is that data used for prediction may fall outside of the training distribution. If the training set was simulated with, for example, a small population density, should we expect the trained network to accurately estimate *σ* if the test data come from a species with a large population density? We set out to explore limitations of disperseNN using deliberately misspecified simulations, including out-of-sample (i) population density, (ii) ancestral population size, (iii) habitat size, and (iv) restricted sampling area relative to the full habitat. We individually address each scenario by augmenting the training set, which ultimately allows us to produce a trained network that performs well across a wide range of these parameter values. This is important because, in practice, we often do not have precise estimates of these parameters.

**Fig. 4.**
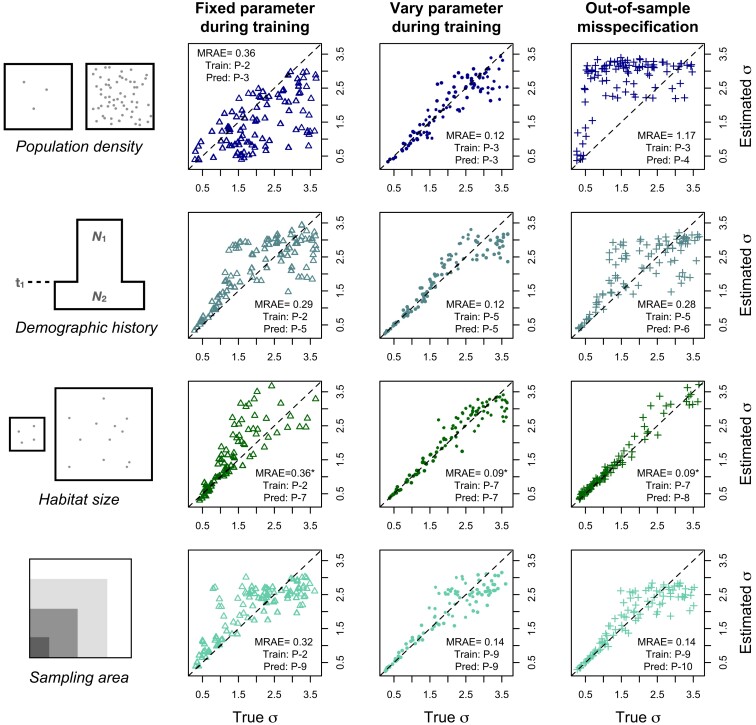
Column 1. Cartoons of unknown parameters that may lead to model misspecification. Column 2. The unknown parameter was fixed during training, but testing was performed on data with different values of the parameter. Column 3. The unknown parameter was *varied* during training, and testing was performed on data from the same distribution. Column 4. The unknown parameter was varied during training, but testing was performed on out-of-sample values, i.e. larger values than were seen during training. The dashed lines are y=x. Outliers greater than 3 are excluded from the fixed-habitat-size plot. “Train: P” and “Pred: P” refer to the Parameter Sets used for training and testing, respectively. MRAE is the mean relative absolute error. All analyses used samples of n=100 individuals. (*The third row has a separate baseline MRAE, 0.09, due to using a smaller carrying capacity, which was chosen to alleviate computation time.)

First, we obtained a baseline level of accuracy by training disperseNN on data where all simulation parameters were fixed except for *σ* (Parameter Set 2). This resulted in an MRAE of 0.12, averaged across test data whose values of *σ* were drawn from the same distribution as the training set (and other parameters were the same). We next used the model trained on Parameter Set 2 to estimate *σ* in test data where one of the aforementioned variables is drawn randomly from a distribution, and thus misspecified (i.e. differing from the value used for training simulations) to varying degrees (Parameter Sets 3, 5, 7, 9). Such model misspecification reduced the accuracy of *σ* estimation (Fig. 4, first column of plots). This reduction in accuracy was most pronounced for misspecified population density and habitat width (MRAE =0.36 for each). Note that effective population density is an emergent property of the simulation that depends on many aspects of demography; in this experiment, we only varied “carrying capacity” (a parameter that affects survivorship), but we expect demographies that are misspecified in other ways (e.g. varying fecundity) to show qualitatively similar results. As shown in Fig. 4, the other scenarios also increased error, although more moderately. When a fixed habitat width was assumed, 23% of predictions were larger than the maximum *σ* from training; for other nuisance parameters all predictions fell within the range of *σ* used in training. Rousset’s method suffered from similar increases in error when run on the same misspecified test data as disperseNN (Table 3). IBD-Analysis was robust to demographic history, consistent with the findings of Ringbauer *et al.* (2017), however, it had issues with the smaller population densities encountered in Parameter Set 3.

**Table 3.** MRAE from misspecification experiments using three different inference methods.

Treatment	Parameter set	disperseNN	Rousset	IBD-Analysis
Baseline	2	0.12	0.14	0.09
Density	3	0.36, 0.12	0.55	0.20
Demographic history	5	0.29, 0.12	0.39	0.10
Habitat size	7	0.36, 0.09^*a*^	0.44	0.11
Sampling area	9	0.32, 0.14	0.46	0.16
Point sampling	2	0.20, 0.12^*b*^	0.59	0.29
Asymmetric sampling	2	0.14, 0.12	0.19	0.10
Transect sampling	2	0.25, 0.09	0.20	0.09
Hourglass habitat	2	0.33, 0.09	0.69	0.17
C-shaped habitat	2	0.22, 0.09	0.51	0.20

The disperseNN program was trained using Parameter Set 2 and applied to test data from the parameter set listed in the second column. The Rousset and IBD-Analysis methods were run on the same test data as disperseNN, except IBD-Analysis used true identity-by-descent blocks; thus, the MRAE is not comparable between IBD-Analysis and the other methods which used only 5,000 SNPs. Two values are shown for disperseNN: error with misspecified (left) or correctly specified (right) model; the latter was trained while varying the corresponding nuisance parameter, or trained with a sampling strategy and habitat shape that reflect the test data.

^*a*^This experiment used a smaller carrying capacity than the others, therefore the disperseNN “baseline” MRAE for variable habitat size was 0.09 instead of 0.12.

^*b*^This experiment used a smaller sample size, n=91, which led to a baseline MRAE of 0.13.

Having observed the effect of misspecification, we next assessed how well the problem could be ameliorated by training across a range of plausible values instead of at a single, fixed value. To do this, for each of the four parameters we train a model using simulations in which the parameter is drawn from a distribution (and, in fact, we reuse Parameter Sets 3, 5, 7, 9 for this purpose). In each case, disperseNN learned to accurately estimate *σ* when individual nuisance parameters were unknown, with error levels approaching the original MRAE (Fig. 4, column 3; Parameter Sets 3, 5, 7, 9). To reiterate, in this procedure, we carried out four experiments, varying a single unknown parameter at a time, not in combination. Essentially by treating each unknown parameter as a nuisance parameter during training, the model can become agnostic to the unknown parameter—or else learn a representation for the parameter such that *σ* can be calculated conditional on the learned parameter. This ability is critical for applying supervised learning methods for estimating *σ* where model parameters other than *σ* are unknown.

Although disperseNN was able to predict *σ* after including variation in each nuisance parameter in the training set, we next show that extrapolation is limited in some cases for unfamiliar parameter values, i.e. values outside of the distribution used for training. In the preceding trial, the same distribution was used for both training and prediction. Next, we assessed disperseNN’s ability to extrapolate at very large values of each nuisance parameter (Parameter Sets 4, 6, 8, 10), beyond the range used in training (Parameter Sets 3, 5, 7, 9). Results from this experiment were varied (Fig. 4, rightmost column): predictions at out-of-sample values of density and ancestral population size were unreliable, but we were able to predict at large, out-of-sample habitat sizes and sampling areas quite well. It is noteworthy that using very large habitat sizes resulted in only a single estimate being 1% larger than the maximum *σ* from training.

Another variable that affects the performance of disperseNN is sampling strategy (Table 3; Supplementary Fig. S9). While in the preceding experiments, genotypes were obtained from individuals sampled uniformly at random, real data rarely approximate a uniform sample. We found that after training with uniform sampling, disperseNN predictions were somewhat less accurate when given data sampled in clusters or sampled more heavily from one-half of the habitat. A more substantial error was introduced with transect sampling. However, once disperseNN was trained using each of the alternative sampling strategies, accuracy was restored to baseline levels or became even more accurate than uniform sampling (Table 3). In light of this, we advise practitioners to build the empirical sampling strategy into the training simulations for disperseNN. In addition, if the shape of the habitat is misspecified during training, predictions from disperseNN and the other methods will be skewed (Supplementary Fig. S10; Table 3); however, note that this scenario has additional complexity, because it also includes misspecified habitat *area* (i.e. population size) and misspecified sampling distribution. It is important to consider these complications when designing training simulations for disperseNN to ensure accurate predictions. However, unlike existing methods, disperseNN has the potential to address these issues during training.

### Training with several unknown parameters

**Fig. 5.**
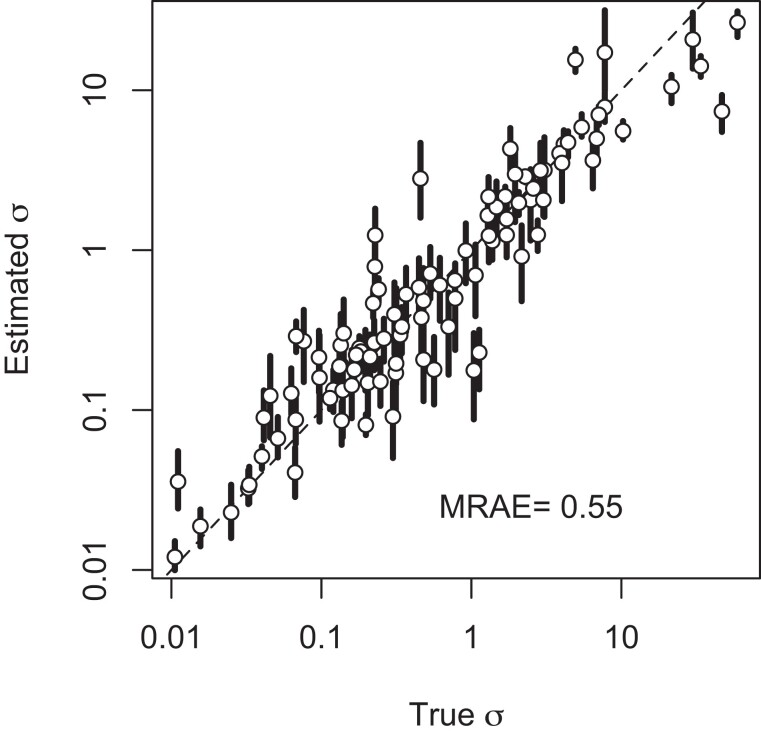
Validation of the pretrained model (Parameter Set 11). Shown are 100 test datasets, each generated from an independent simulation. Open points indicate the mean estimate from 1,000 subsamples of 5,000 SNPs drawn from each dataset, with the sample size varying uniformly between 10 and 100 for each subsample. Also depicted is the range of estimates from the middle 95% of subsamples. The dashed line is y=x. Note the log scale. MRAE is the mean relative absolute error.

We next sought to train a network that could estimate *σ* even when multiple nuisance parameters are unknown. The resulting network is what we refer to as “the pretrained network.” To do this, we used large ranges for parameters that control: (i) dispersal distance, (ii) population density, (iii) ancestral population size, (iv) timing of population size change, (v) habitat size, and (vi) the size of the sampling area relative to the full habitat (Parameter Set 11). Furthermore, we exposed disperseNN to a range of different sample sizes between 10 and 100 by padding the genotype matrix out to 100 columns during training. However, in all cases, sampled individuals were chosen uniformly at random. Training simulations used 5,000 SNPs sampled from a single 100 megabase chromosome with recombination rate $10^{-8}$; this approach resembles a RADseq experiment, as the loci are spaced out on the chromosome and may be considered mostly unlinked. Last, we collapsed the diploid genotypes output by SLiM into unphased genotypes: 0s, 1s, and 2s; representing the count of the minor allele at each variable site. Through validation with held-out, simulated data, we found that the final model could predict *σ* reasonably well across a wide range of nuisance parameter values (MRAE=0.55; Fig. 5). For comparison, we ran Rousset’s method (with density misspecified as D=1, which is close to the mean of the training simulations) and IBD-Analysis (with true identity by descent blocks) on the test data from Parameter Set 11, which gave MRAE=32.26 with 24% undefined and MRAE=2.44 with 2% undefined, respectively.

We provide the learned weights and biases from the above pretrained network for download as part of the disperseNN package. The pretrained network can be used to quickly estimate *σ* from various species or simulated datasets without additional training or simulations. We note that the pretrained network for disperseNN could, in addition, be an excellent starting place for transfer learning (Weiss *et al.* 2016) for specific organisms, sampling designs, or perhaps alternative datatypes (e.g. microsatellite mutations). In our system, the pretrained network took 6.5 s to estimate *σ* using a dataset of 10 individuals and 5,000 variants, with the majority of computation time spent loading software libraries and preprocessing the genotype matrix.

The pretrained model will be more appropriate for some datasets than others. First, the model was trained on 10–100 individuals sampled across a region of known width. Therefore, data collected from a single location are not expected to give accurate predictions (unless the breeding locations were known and spatially distributed). While disperseNN can be trained with any number of SNPs, the pretrained network uses 5,000. Therefore, if fewer than 5,000 variants are available, as in some RADseq datasets, then a new network must be trained to match the empirical number. Padding the input genotypes with zeros will not suffice for using fewer SNPs, as we did not train with zero-padding. Although we aimed to produce a pretrained model that is widely applicable, many of the attempted simulations either resulted in population extinction, or could not be simulated due to computational constraints, which resulted in parts of parameter space not represented in the realized, multivariate training distribution (Supplementary Fig. S5). Therefore, we expect this model to be most applicable for smaller populations that fall solidly inside of the training distribution. See Parameter Set 11 in the Materials and Methods section for “prior” ranges.

Additional training will be beneficial in some situations. If independent estimates for nuisance parameters or better-informed “prior” ranges are available, new training data may be tailored using the better-informed values. Species range maps with detailed geographic boundaries can be simulated with SLiM (since version 3.5) or slendr (Petr *et al.* 2022), which could be superior to the square map we used. Importantly, if empirical parameters fall outside of the training distributions used for the pretrained network, e.g. very large sampling area, then new training data will need to be generated that reflect the real data.

### Quantifying uncertainty

In addition to helping us generate training data, simulation also allows us to quantify uncertainty through validating our models on held-out test datasets. Indeed, our reported values of MRAE give a sense of how much error to expect when applying the method to real data, in so far as the data resemble a typical draw from our test simulations. For example, in the above experiments that included one or zero nuisance parameters, the MRAE from in-sample tests was on the order of 0.12. Therefore, using a model with MRAE of 0.12, we might expect future predictions to be off from the true values by about 12%. However, if the real data are not well represented by the simulations, for example, if the density of the analyzed population does not resemble that of the training simulations, then predictions might be less accurate, or biased.

Since we get distinct estimates for each subset of *m* SNPS, we can also assess uncertainty by looking at the range of variation among these estimates, i.e. through nonparametric bootstrapping. Each subsample of *m* SNPs from the same set of sampled individuals gives a different estimate of *σ* because of the varying genealogical histories that underlie different subsets of genomic loci, so the range of variation reflects the uncertainty arising from this genealogical noise. However, note that the bootstrapped estimates are not independent, because of linkage and because they come from a single set of individuals. The disperseNN program provides a built-in functionality for performing this bootstrapping procedure, and will report the distribution of estimates across replicate draws of *m* SNPs (each draw is made without replacement from the complete set of available SNPs, but the replicates are drawn independently and so may overlap).

Although the distribution of these estimates should reflect uncertainty somehow, it is not immediately clear how to convert this into a formal quantification of uncertainty. This distribution of estimates is not a sample from a well-calibrated posterior distribution (nor should we expect it to be): in the test data for the pretrained model (Fig. 5), the true *σ* was covered by the middle 95% range from the bootstrap distribution for only 51% of simulated datasets. However, we can inflate the interval obtained by a scalar value such that our bootstrap interval is better calibrated. On our validation set for the pretrained model, this scalar value is 3.8, which leads to intervals that cover the true value for 95% of our test simulations. (If $\hat{\sigma }$ is the mean of the bootstrap estimates, and *a* and *b* are the 2.5% and 97.5% quantiles, respectively, then the resulting interval is from $\hat{\sigma }-3.8(\hat{\sigma }-a)$ to $\hat{\sigma }+3.8(b-\hat{\sigma })$.) However, if this is to be a recipe for a well-calibrated credible (or, confidence) interval, then it needs to apply regardless of the situation: i.e. the magnitude of the error should be a roughly constant multiple of the range of the bootstrap estimates. Happily, this seems to be the case: in our experiments, we found the error to be roughly a constant multiple of the width of the range of bootstrap estimates. Concretely, if *σ* is the true value, $\hat{\sigma }$ is the estimated value, and *w* is the range of values from 100 bootstrap estimates, then $|\sigma -\hat{\sigma }|/w$ has no significant associations with any of the model parameters using Parameter Set 11; see Supplementary Fig. S11.

In summary, this suggests that the middle 95% interval of bootstrap estimates, inflated by a factor of 3.8, can stand in for a 95% credible interval for results obtained from our pretrained neural network. Of course, since this is an empirically derived result, we cannot guarantee the same inflation value to be appropriate for other networks or for datasets not well-represented by the simulations in training set for our pretrained model.

### Empirical findings

We used disperseNN to estimate *σ* from a diverse set of organisms using preexisting empirical datasets. The pretrained disperseNN model works with a wide range of genetic data, including low-coverage whole genome sequencing or RADseq data, because genotypes were not phased during training. Deviations in mutation rate between the training and empirical data will not affect the results, because we trained disperseNN with a fixed number of SNPs—5,000—sampled throughout the genome. Likewise, deviations in recombination rate are unlikely to be a concern because we created the pretrained model with SNPs that are fairly spaced out across the genome and are mostly independent, and the empirical SNPs are similarly distributed. For some empirical datasets, we analyzed a subset of sample localities in order to keep the sampling width less than 1,000 km; accordingly, we report sample sizes and sampling widths from the subsampled region, rather than the full dataset. Also, we ensure that each sample location is represented by only one individual. For each dataset, we used disperseNN to prepare the two inputs for the CNN: the SNP table was converted to a genotype matrix and the distance between the furthest individuals was calculated. Next, we used disperseNN to predict *σ* on each of 1,000 independent bootstrapped samples of 5,000 SNPs, obtaining a distribution of *σ* estimates. Table 4 shows the mean and approximate 95% credible interval of *σ* estimates, along with other analysis parameters, for each empirical dataset. We note, however, that the credible intervals for empirical datasets with smaller numbers of SNPs may be poorly calibrated (e.g. *Peromyscus leucopus*, that has only slightly more than 5,000 SNPs).

**Table 4.** Empirical results.

Species	Common name	Region	*σ* (km)	95% CI (km)	Previous (km)	$N_{\mathrm{loc}}$	*n*	*S* (km)	M. dist.
*Zosterops borbonicus*	Réunion grey white-eye	Réunion	4.97	(1.76, 13.83)	NA	295	41	62	4.59
*Peromyscus leucopus*	White-footed mouse	New York	0.77	(0.32, 1.67)	0.03–0.11	Undef.	12	38	8.15
*Anopheles gambiae*	African malaria mosquito	Cameroon	10.29	(2.00, 48.03)	0.04–1.57	52	29	278	9.62
*Bombus bifarius*	Two-form bumble bee	Washington	14.75	(5.60, 37.28)	1.2–5	1,147	14	273	10.47
*Bombus vosnesenskii*	Yellow-faced bumble bee	California	7.70	(1.21, 38.11)	1.2–5	3,944	18	169	11.83
*Hippoglossus hippoglossus*	Atlantic halibut	Canada	4.29	(0.71, 33.85)	NA	Undef.	11	193	14.59
*Crassostrea virginica*	Eastern oyster	Canada	1.52	(0.72, 4.31)	21.9	1,435	13	187	19.69
*Canis lupus*	Grey wolf	N. America	15.68	(2.36, 107.3)	98-147	35	13	721	25.42
*Helianthus petiolaris*	Prairie sunflower	Kansas	1.00	(0.39, 3.52)	0.156	9	11	204	45.28
*Zosterops olivaceus*	Réunion olive white-eye	Réunion	1.05	(0.27, 4.36)	NA	2,392	10	50	45.97
*Helianthus argophyllus*	Silverleaf sunflower	Texas	1.04	(0.38, 4.08)	0.156	57	30	307	86.49
*Arabidopsis thaliana*	Thale cress	Spain	1.36	(0.28, 5.05)	0.001	35	35	80	198.25
*Arabidopsis thaliana*	Thale cress	Sweden	0.44	(0.20, 0.93)	0.001	84	84	325	428.17

The *σ* column is the mean from 1,000 subsamples of 5,000 SNPs. “95% CI” is the credible interval obtained from bootstrapping. The “Previous” column shows previously published estimates for dispersal distance. $N_{\mathrm{loc}}$ is the neighborhood size using the Rousset calculation. In other columns, *n* is sample size, *S* is the width of the sampling area in kilometers, and “M. dist.” is the Mahalonobis distance from the center of the training distribution with respect to five summary statistics: nucleotide diversity, Tajima’s D, inbreeding coefficient, observed heterozygosity, and expected heterozygosity.

When available, we report previous dispersal estimates from the literature. Independent estimates came from a variety of methods, including mark-recapture, tracking devices, and the Rousset method. Overall, we find a correlation ($r^{2}=0.39$; p=0.03; linear regression on log-transformed inputs) between our estimates and previous estimates using different methods. We might expect each of the analyzed empirical datasets to deviate from our training set in some way. To get a rough estimate of the “distance” between an empirical dataset and our training set, we calculated five summary statistics—nucleotide diversity, Tajima’s D, $F_{IS}$ (an estimate of inbreeding), observed heterozygosity, and expected heterozygosity—and calculated the Mahalanobis distance between the centroid of the training distribution and each dataset, according to: $D^{2}=(x-m{)}^{T}\cdot C^{-1}\cdot (x-m)$, where $D^{2}$ is the Mahalanobis distance squared, *x* is a vector of summary statistics from an empirical dataset, and *m* and *C* are the vector of means and the empirical covariance matrix of the summary statistics in the training data. Thus, smaller distances have summary statistics more similar to the training distribution, and distances larger than roughly 40 fall outside of the training distribution (Supplementary Fig. S12). However, a small Mahalanobis distance does not guarantee that the model is well-specified for a given dataset. In fact, we expect error to accumulate due to misspecified sampling scheme, irregular habitat, and other variables that affect population genetic variation, some of which are mentioned below.

*Zosterops*: Réunion grey white-eye and Réunion olive white-eye are endemic to the island of Réunion with approximate land area of 2,500 km^2^. These species’ restricted ranges make them ideal for analyzing with our pretrained model. We analyzed the RADseq data from Gabrielli *et al.* (2020) including 41 individuals and 7,657 SNPs from *Z. borbonicus* and 10 individuals and 6,103 SNPs from *Z. olivaceus*. Our estimate for *Z. borbonicus* was 5.0 km, however the estimate in *Z. olivaceus* was smaller, 1.1 km. Although we are not aware of other dispersal estimates in these species, the data curated by Paradis *et al.* (1998) include natal dispersal estimates for 75 birds, and the smaller species, comparable in size to *Zosterops*, have dispersal distances in the range of 1–20 km. The mean estimates for both species fall within the range from Paradis *et al.* While the data for both *Zosterops* species are similar, summary statistics in *Z. olivaceus* were further from the centroid of the training distribution.

*Peromyscus leucopus*: From the white-footed mouse RADseq dataset of Munshi-South *et al.* (2016), we analyzed 12 individuals collected from the New York City metropolitan area, with 5,536 SNPs. We estimated dispersal distance to be 770 m. For comparison, Keane (1990) and Jacquot and Vessey (1995) measured natal dispersal in white-footed mice in rural locations. They reported mean dispersal of 85–109 m in males and 25–88 m in females, which are smaller than our estimate. However, their estimates are likely constrained to some degree by the small study areas used for recapture. Indeed not all mice were recaptured in Jacquot and Vessey (1995), leaving open the possibility of long-distance movements outside of the study area. For example, Murie and Murie (1931) documented travel distances greater than 1 km in *Peromyscus maniculatus*. Occasional long-distance dispersal may help reconcile the difference between previous estimates and ours.

*Anopheles gambiae*: From the whole genome resequencing dataset from the *Anopheles gambiae* 1000 Genome Consortium (2021), we analyzed 29 individuals with 11 million SNPs. Our estimate in *A. gambiae* of 10.3 km is substantially larger than mark-recapture estimates. For comparison, Epopa *et al.* (2017) measured individual *A. coluzzii* dispersal distances between 40 and 549 m over seven days; however, the geographic study region was restricted to a single village. Another study, Gillies (1961), reported mean dispersions of 837–1,577 m, depending on sex and release location. A third study reported *per-day* movements of 350–650 m (Costantini *et al.* 1996). It is unclear to what degree long-distance dispersal in mosquitos contributes to effective dispersal and gene flow. Remarkably, the recent study of Huestis *et al.* (2019) captured *A. gambiae* and other mosquito species 40–290 m above the ground, suggesting a wind-borne dispersal mechanism. Assuming average wind speeds, Huestes *et al.* estimated that each year tens of thousands of *A. gambiae* individuals migrate 10 or 100 s of km in the atmosphere of the studied region. These findings suggest that dispersal potential in this species is considerably larger than once thought. Significant long-range dispersal in *A. gambiae* is consistent with some predictions in the species, as there is little genetic differentiation across portions of the species range (e.g. West Africa), while at broader scales structure is appreciable (Anopheles gambiae 1000 Genome Consortium, 2017).

*Bombus*: From the dataset of Jackson *et al.* (2018), we examined RADseq data from two bumble bee species, *B. bifarius* and *B. vosnesenskii* with samples sizes of 14 and 18, and 8,073 and 6,725 SNPs, respectively. Our estimated dispersal distances were 14.8 and 7.7 km for the two. These species are eusocial, so our dispersal estimate should reflect the distance traveled by queens that start successful nests. Mark-recapture analyses have found a minimum distance traveled by queens in other *Bombus* species of 1.2 km (Carvell *et al.* 2017), and using genetic full-sib reconstruction resulted in 3–5 km (Lepais *et al.* 2010). These estimates are particularly relevant, as they measure natal dispersal from the birth location of the queen. Even so, these values represent a lower bound distance that queens disperse, as there was potential for longer-distance dispersal events that fall outside of the study area. Our results may offer a glimpse into bumble bee dispersal, including longer distances that would be difficult to measure directly.

*Hippoglossus hippoglossus*: From the RADseq data of Kess *et al.* (2021), we analyzed 11 individuals with 69,000 SNPs. Tagging studies find mean halibut movements greater than 100 km (Liu *et al.* 2019). However, the distance traveled by adults in search of food may be considerably larger than the quantity we wish to estimate which is proportional to the mean distance between the birth location and parental birth location. Indeed, there is spatial structure distinguishing Atlantic halibut stocks due to spawning site fidelity (Shackell *et al.* 2021). Therefore, the observed sample locations—used to calculate the second input to disperseNN—are likely foraging locations that may differ significantly from the breeding locations. However, if assumptions about the size of the spawning area can be made, disperseNN provides a novel approach for inferring effective *σ* in foraging or migrating individuals for whom “home” locations are not known. Our estimate of 4.3 km (using the sampling width as the second input) could be close to the true dispersal distance if birth site fidelity is quite high. In another large marine species, *Diplodus sargus sargus*, natal dispersal distance was measured to be 11 km using otolith chemistry (Di Franco *et al.* 2012).

*Crassostrea virginica*: From the RADseq data of Bernatchez *et al.* (2019), we analyzed 13 individual eastern oysters with 7,097 SNPs. This species has larval dispersal (Vercaemer *et al.* 2010) and occasional adult translocations (Bernatchez *et al.* 2019). Our estimate of 1.5 km is much smaller than the previous estimate of 21.9 km (Rose *et al.* 2006). We offer several possible explanations for this discrepancy. We expect that oyster dispersal is at least in part passive, particularly during the larval stage, and so depends on local currents. The previous estimate was from a different sample region, Chesapeake Bay, which likely has different local conditions than the coast of Canada where the samples that we analyzed were collected. Second, the previous estimate used microsatellite loci to estimate density in order to implement the Rousset method. Density is notoriously difficult to estimate from genetic data, so it would not be surprising if this step contributed to error. In contrast, disperseNN is designed to work around the unknown density parameter. However, we note that the range of this species is likely to be closer to a narrow strip than the broad square or rectangle used in our simulations.

*Canis lupus*: From the RADseq dataset of Schweizer *et al.* (2016), we analyzed data from 13 individual wolves genotyped at 22,000 SNPs. Exceptionally good data exist on wolf dispersal from radio collars. A commonly reported value for this species is the distance traveled by adults that disperse between territories. For example, some estimates for this value include 98.1 km (Jimenez *et al.* 2017), 98.5 km (Kojola *et al.* 2006), and 147.0 km (Barry *et al.* 2020). However, not all individuals disperse from their natal territory. For example, Kojola *et al.* (2006) and Barry *et al.* (2020) reported that 50% and 47% of individuals dispersed between territories, respectively. Jimenez *et al.* (2017) reported more nuanced statistics: 18% of collared individuals had documented dispersal, survival was lower in dispersers, and not all dispersers reproduced. It is unclear how frequent breeding occurs *within* the natal pack; if 85–90% of reproduction occurred without movement between territories, then our estimate of 15.7 km might be reasonably close to the true, effective dispersal distance.

*Helianthus* : We analyzed two wild sunflower species from Todesco *et al.* (2020): *Helianthus petiolaris* (n=11; 61,000 SNPs) and *H. argophyllus* (n=30; 60,000 SNPs), with whole genome resequencing data. Wild sunflowers regularly outcross, therefore, the estimated *σ* in part reflects pollinator distance, in addition to transport of seeds by animals and other methods. Previously, Arias and Rieseberg (1994) reported the frequency of hybridization between cultivated and wild sunflowers at distances between 3 and 1000 m; if we convert these hybridization frequencies to counts of hybridization events, the mean distance of these pollination events was 156 m. The estimates from disperseNN were larger: 1,000 and 1,040 m in *H. petiolaris* and *H. argophyllus*, respectively. These estimates seem large but may be reasonable if pollination occurs via bees, which can have foraging ranges greater than 1 km (Visscher and Seeley 1982; Osborne *et al.* 2008). Studying foraging distance in pollinators is an active area of research, however, Pasquet *et al.* (2008) used an exceptionally large study area and radio trackers to find a median flight distance of 720 m in carpenter bees. Our estimates for the two analyzed *Helianthus* species were similar to each other.

*Arabidopsis thaliana* : From the whole genome resequencing dataset of the The 1001 Genomes Consortium (2016), we analyzed two sampling clusters from different geographic regions: Spain (142,000 SNPs, n=35) and Sweden (124,000 SNPs, n=84). Our *σ* estimates from these two groups of samples were 1,360 and 440 m, which are considerably larger than the average distance that seeds fall from the parent plant; Wender *et al.* (2005) estimated that the average distance traveled by *A. thaliana* seeds with wind are less than 2 m. However, occasional long-distance seed dispersal, e.g. via water or animals, and infrequent outcrossing via insect pollination may inflate the effective dispersal distance in this species. The outcrossing rate of *A. thaliana* has been estimated to be $3\times 10^{-3}$ (Abbott and Gomes 1989). Importantly, *A. thaliana* is predominantly selfing and the analyzed samples are (naturally) inbred, while our training set which did not include selfing. *A. thaliana* has experienced a known population expansion (Tyagi *et al.* 2016), and although we attempted to account for demographic history during training the true history of *A. thaliana* may not be well-represented by our simplistic range of population histories. There was a three-fold difference in estimated dispersal distance between the analyzed populations, perhaps due to local environmental differences between Spain and Sweden or different pollinator species.

## Discussion

### Dispersal estimation using deep learning

Understanding how organisms move across land or seascapes is critical for gaining a full picture of the forces shaping genetic variation (Wright 1943; Kimura and Weiss 1964; Barton *et al.* 2002). However, it remains difficult to confidently infer spatial population genetic parameters. Here, we present a deep learning framework, disperseNN, for estimating the mean per-generation dispersal distance from population genetic data. There are several advantages of our method over existing population-genetics-based estimators, including improved accuracy for small (n=10) to moderate (n=100) sample sizes, accessible input data (unphased SNPs), and the ability to infer dispersal distance in the face of unknown model parameters such as population density. It is unclear how competitive disperseNN would be with other programs if given very large sample sizes (e.g. thousands of genotypes), because the memory requirements for such a scale may be limiting. These improvements open the door for using DNA to infer dispersal distance in nonmodel organisms where population density is unknown or identity-by-descent tracts are out of reach. Perhaps more importantly, because disperseNN uses a form of simulation-based inference, analyses do not depend on idealized mathematical models of flat, featureless space, and can be tailored for the particular study system, for instance, detailed habitat maps and independent estimates for key model parameters can be readily incorporated.

Unlike previous genetics-based estimators that use geographic distances between individuals, our neural network does not see the relative spatial locations of individuals. This means that our neural network could in theory be applied to genetic data for which sampling locations are unavailable due to ethical or legal protections, or applied to adult individuals that have ranged far from their nesting or spawning area. However, to do so an estimate of the sampling width is required as input by disperseNN. We had initially sought to convey the spatial sampling coordinates to the network, inspired by Rousset (1997) and Ringbauer *et al.* (2017), however doing so did not improve the model’s accuracy. This surprising result may have to do with limitations of the chosen architecture. Or, perhaps the spatial coordinates convey little additional information for the task we are interested in beyond the signal inherent to the genotypes. disperseNN is able to infer dispersal without individual sample locations, because the dispersal rate affects not only pairwise genetic distances between individuals, but also population genetic variation more generally, such as the site frequency spectrum and its transforms (e.g. nucleotide diversity), inbreeding coefficients, and linkage disequilibrium (Battey *et al.* 2020b). Additionally, it is well-known that genotypes can often be used to obtain a reasonably good estimate of relative sampling locations (e.g. by PCA; Novembre *et al.* 2008). disperseNN, by using a CNN with a genotype matrix as its input, is able to capture population genetic information from raw data as has been seen in a few prior contexts (e.g. Flagel *et al.* 2019; Gower *et al.* 2021; Sanchez *et al.* 2021).

Another strength of the deep learning approach is its versatility. In particular, disperseNN can be used with unphased SNPs and small sample sizes, which makes it applicable for a variety of genomic dataset types. In contrast, recently developed tools for dispersal estimation require identity-by-descent blocks as input (Ringbauer *et al.* 2017; Al-Asadi *et al.* 2019). Although these methods perform well when high-quality data are available, phasing and identity-by-descent inference in nonhuman genomes is a considerable challenge, especially for RADseq. Unphased SNPs, on the other hand, are more widely available.

Next, disperseNN can infer dispersal without a priori knowledge of other important parameters, such as population density. In contrast, the commonly used Rousset method requires an independent estimate for population density in order to infer dispersal distance. Our supervised learning approach can learn to predict *σ* in the face of unknown density, which is achieved by exposing the network to training datasets with various densities. Through this procedure, disperseNN successfully learned to estimate *σ* in test datasets regardless of density, conditioned on true density being within the training distribution. While that is so, we still observed misspecification for large, out-of-distribution densities, which caused the network to overestimate *σ*. We used the same approach to deal with uncertainty of various other parameters. On the other hand, if independent estimates for some parameters or better-informed “priors” are available, then training can be customized to reflect the known parameters. It is worth mentioning that our general inference framework can be easily modified to infer multiple parameters at once, namely population density and dispersal, although we have not explored this yet.

Thus far we have focused on the indirect estimation of dispersal distance, without measurements of how far individuals move. For a review of other genetic techniques for estimating dispersal distance, including direct and indirect methods, see Broquet and Petit (2009). Recently, two studies have used close kin mark-recapture approaches for estimating dispersal distance, which were applied to mosquito species (Jasper *et al.* 2019; Filipović *et al.* 2020). Close kin mark-recapture uses the genome of a close relative to represent a “recapture,” thereby skipping the need to physically recapture individuals. These promising new methods estimate dispersal distance by modeling the spatial distribution of close kin. In theory, our approach may offer advantages over close kin mark recapture: disperseNN aims to estimate *effective* dispersal, has no requirement for close kin to be captured together, and works with small sample sizes (n=10). The ability to capture kin relies on a sample size that is a sufficiently large proportion of the local population size, which is not always feasible.

### Limitations

Although training on simulated data allows great flexibility, the simulation step was also a limitation for the current study. In particular, generating the training data for our pretrained network involved very long computational run times and large memory requirements: up to 175 gigabytes of RAM and two weeks of run time for the largest parameterizations of individual simulations. Shortcuts were used to reduce simulation time, including running fewer spatially explicit generations, and sampling multiple times from each simulated population (see Materials and Methods). Of course, if new training data are generated for a population that is comparatively small, then the simulation burden will be smaller.

As with many statistical approaches, disperseNN has limited ability to generalize outside of the range of parameter values on which it was trained. Although exposing the model to training datasets with varying parameter values successfully produced estimates robust to variation in those parameters, the resulting models were still unable to provide good estimates for out-of-sample data. For instance, if the test data came from a population with density higher than those the network had seen during training, *σ* was overestimated. Likewise, prediction error increased if the test data had a larger spatial sampling area than the network saw during training. Therefore, we expect the pretrained model from our empirical analysis to be most accurate for smaller spatial samples from smaller populations—parameters that fall inside the training range—while applications to larger populations may be more questionable. In fact, it is generally recommended to restrict the sampling area to a small region when estimating *σ* to avoid issues with environmental heterogeneity and patchy habitats (Broquet and Petit 2009; Shipham *et al.* 2013). However, a larger sampling area is clearly required to infer a large *σ*.

Another potential issue with our approach is complex demographic history. As demographic perturbations leave a footprint in contemporary genetic variation, demography may bias estimates of *σ* for a neural network trained with a particular history, e.g. constant size. This issue is by no means unique to our analysis. Leblois *et al.* (2004) showed that dispersal rate inference using Rousset’s technique was affected by past demographic values rather than recent population density. We attempted to address this in our analysis, by simulating under random two-epoch models. This approach produced accurate estimates for test data with a similar two-epoch history. However, it also suggests that different, more complex demography may reduce accuracy, for example, a more extreme bottleneck than was simulated in training, fluctuating size, pulse admixture, or perhaps population structure not captured in our simulations (e.g. barriers to dispersal or range expansion). Identity-by-descent-based methods may alleviate the effect of ancestral population structure because long identity-by-descent tracts originate from the recent past (Barton *et al.* 2013). Similar to demographic history, other model misspecifications such as complex habitats and environmental heterogeneity could also be sources of error for estimation using our method.

Likewise, in our model demography is uniform across space. This assumption may be nearly true—or, at least useful—for certain applications, particularly if the sampling area is small. However, in reality, we expect dispersal to vary across space due to a variety of reasons: for example, mountain ranges will prohibit dispersal for many species. Alternatively, suitable habitat is often discontinuous, and dispersal between patches may be different than within patches. Likewise, heterogeneous habitat can generate source-sink dynamics. Existing methods do infer heterogeneous dispersal surfaces across space (Petkova *et al.* 2016; Al-Asadi *et al.* 2019), but have limitations including (i) estimating relative differences in dispersal as opposed to the magnitude of dispersal, or (ii) requiring identity-by-descent data as input.

When we included multiple nuisance parameters (Fig. 5; Parameter Set 11), the MRAE was larger than that of experiments with only one or zero nuisance parameters (Fig. 4; e.g. Parameter Set 3). This difference can be partly explained by the larger number of parameters with potential to confound. In addition, the *range* of values explored for *σ*, as well as for nuisance parameters, were orders of magnitude larger than those of the other experiments. Finally, it is important to note that in our simulations, a single parameter was used for typical “mother-child” dispersal distances, “mother-father” mating distances, and the “interaction distance” over which population regulation occurs. (However, the quantity we estimate is mean parent-offspring distance, which incorporates the first two.) If these quantities are very different in a real population, the pretrained network may be less reliable than we have estimated.

### Interpretation of empirical findings

We estimated *σ* in a diverse set of organisms using publicly available datasets. These included data obtained by both whole genome shotgun sequencing and RADseq—i.e. variations on standard RADseq (Baird *et al.* 2008) or genotyping-by-sequencing protocols (Elshire *et al.* 2011). Rather than simulate scenarios that would be appropriate to each species independently, we trained a single disperseNN model designed to estimate *σ* without a priori knowledge of density, ancestral population size, or species range.

The majority of empirical results from disperseNN were sensible, however, our estimates for *A. thaliana*—particularly in the population located in Spain—are likely overestimates, in part due to the lack of selfing in our training simulations. *A. thaliana* also had levels of heterozygosity and inbreeding that were outside the range of values observed in the training set, a feature reflected in the Mahalanobis distances between training and prediction sets. In the future, disperseNN might be better tuned to analyze selfing species, but this would require simulating additional training data and subsequent validation steps.

Our approach led to consistently larger dispersal estimates than mark-recapture experiments. Mark-recapture data were available for three of the analyzed taxa—white-footed mouse, *Bombus*, and *Anopheles*. However, the mark-recapture estimates for *Anopheles* are not ideal, as they represent adult-travel distances instead of parent-offspring distances. In contrast, the measurements from bumble bees (Carvell *et al.* 2017) and mice (Keane 1990; Jacquot and Vessey 1995) are particularly relevant, as they measure the distance traveled by queen bees from the original hive or individual mice between birth location and adult territory. In all three cases, our estimate was larger than the mark-recapture calculation, which suggests either an upward bias in the disperseNN output or underestimation in the mark-recapture estimates. In each mark-recapture study, the geographic recapture area was smaller than the sampling area we provided to disperseNN. It is likely that long-distance dispersers, even if less common, are missed during the recapture step, which would bias the inferred dispersal distance downward in direct, mark-recapture studies.

### Population genetics for spatial ecology

We conclude with a call for increased development and applications of population genetics methods for spatial ecology applications. Dispersal is one of the main factors controlling metapopulation dynamics (Leibold *et al.* 2004), as well as the total population size and whether a population persists (Gadgil 1971). Therefore, dispersal estimates are critical for choosing appropriate settings in population viability analyses (Akçakaya and Brook 2008). Likewise, geographic habitat shifts are ongoing for many species, and species’ survival may thus depend on their ability to disperse fast enough to follow rapidly changing local conditions (Wiens 2016). Thus, obtaining values for dispersal distance are important for species distribution modeling which is used to project future species ranges (Wiens *et al.* 2009). In the comprehensive review of Driscoll *et al.* (2014), the authors present a list of 28 applications for which dispersal values were needed in conservation management, and report several independent calls for improved dispersal information and dispersal inference methods (Sutherland *et al.* 2006; Broquet and Petit 2009; Ceballos *et al.* 2009; Kingsford *et al.* 2009; Noss *et al.* 2009; Pullin *et al.* 2009; Hadley and Betts 2012).

Characterizing dispersal is also important for managing animal populations relevant to human health. For example, in the fight against malaria, we must identify migration corridors and source-sink dynamics in mosquito vector species to allocate pesticide treatment and to predict the spread of genetic variants conveying insecticide resistance (Clarkson *et al.* 2020). Understanding dispersal is particularly important for modeling and implementing gene-drive strategies (North *et al.* 2013, 2019, 2020; Beaghton *et al.* 2016, 2017; Champer *et al.* 2021; Beaghton and Burt 2022) for controlling the spread of mosquito-borne diseases including malaria.

Direct methods such as radio tracking or genetic identification may provide near-perfect measurements of dispersal within the generation or generations analyzed. However, it is often more valuable to know the expected dispersal distance over many generations, conditional on survival and successful reproduction of the dispersing individuals. For example, the day-to-day foraging distance or seasonal migration distances traveled by adults may differ from the effective dispersal distance. Direct methods such as mark-recapture are often expensive and as a result are limited to relatively small geographic areas, which may ignore long-distance movement and bias the resulting estimate. Therefore, population genetic tools may complement direct methods for improving our understanding of dispersal.

## Supplementary Material

iyad068_Supplementary_Data

## Data Availability

The disperseNN code is available on GitHub at the following link: https://github.com/kr-colab/disperseNN. Supplemental material is available at GENETICS online.

## Appendix: Additionally investigated inputs to the method

This Appendix describes analyses not included in the main document, including strategies that did not work.

### A1. Attempts to use sampling localities

It is intuitive that signal about dispersal might be gleaned from the individual sample locations, as previous population-genetics-based inference methods use sample locations as input. We tried the following strategies for showing the sample locations to the CNN. In each experiment, we modified the neural network architecture to accommodate the sample locations in various ways. Otherwise, the neural network in each experiment closely resembled the architecture described in the main text.

- *Table of locations.* An n×2 array containing the *x* and *y* coordinates was shown to the CNN in a separate input branch (in place of the sampling width input). This input went through a single 128-unit dense layer with ReLu activation before flattening and concatenating with the previous branch.
- *Stored in genotype matrix.* Additional rows in the genotype matrix were used to store the *x* and *y* coordinates for each individual.
- *3-channel array*. A three-dimensional array was used to store (1) the genotypes, (2) *x* coordinates, and (3) *y* coordinates. In the second and third channels, the spatial coordinates were repeated for *m* rows equal to the number of SNPs. Here, the neural network used 1D-convolution and pooling layers, as described in the main text, however, the convolution and pooling layers spanned all three channels simultaneously.
- *2D CNN*. We also tried a variation of the 3-channel-array strategy using 2D-convolution and pooling layers with a 2x2 window.

For each of the above strategies, we trained the neural network in the same manner as the “baseline” model from the misspecification analysis in the main text. The outcome for each was the same: the mean RAE was indistinguishable from the baseline model that does not include sample locations. Moreover, we shuffled the sample locations input, such that each individual has a randomly assigned location, and the output was unchanged. Our interpretation is that the CNN ignores the location data in the experiments attempted thus far, either because the locations are not necessary for estimating *σ*, or because we failed to effectively show the network the locations.

### A2. Including isolation-by-distance summary statistics

We tested whether isolation by distance information in the form of summary statistics would improve inference of *σ*. Specifically, we summarized isolation-by-distance as:

- *b*, the slope of the line of best fit to genetic distances versus geographic distances.
- $r^{2}$ , the coefficient of correlation between genetic distance and geographic distance.

Including either (or both) of these statistics as a separate input branch of size one (or two) marginally improved validation accuracy. The new input branch went through a 128-unit dense layer with ReLu activation before concatenating with the previous branch. Thus, future empirical applications might explore using the above or different summary statistics alongside the genotype matrix for estimating *σ*, or other population genetic parameters. We did not present these results in the main text because (1) the benefit was negligible, and (2) it is beyond the scope of our study to decide on the most relevant and appropriate summary statistics, as countless other statistics might be evaluated for use with, or without, the genotype matrix that we used.
