# Supplementary material for: Dispersal inference from population genetic variation using a convolutional neural network
Source: Genetics. 2023 Apr 13;224(2):iyad068. doi: 10.1093/genetics/iyad068 (PMC10213498; doi:10.1093/genetics/iyad068)
Supplement: iyad068_Supplementary_Data [file iyad068_supplementary_data.pdf]

## Supplementary material

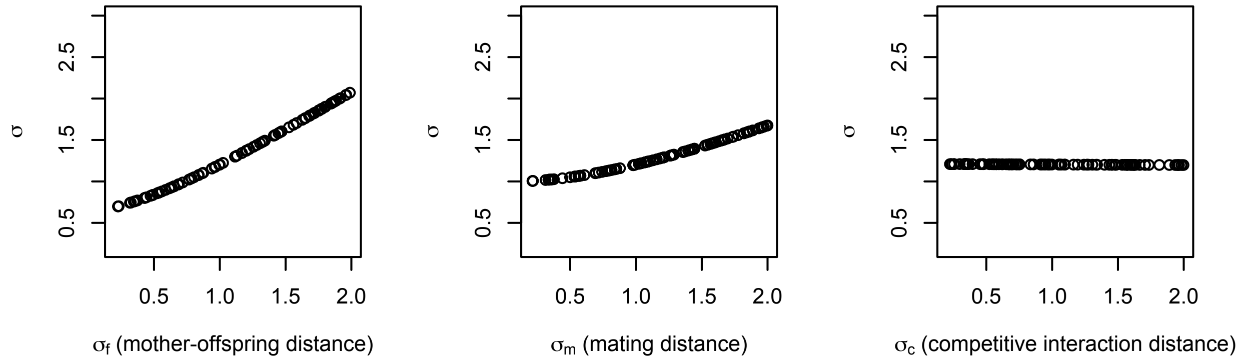


**Figure S1**. Visualizing true $\sigma$, while varying one of three spatial interaction distances at a time: mother-offspring distance ($\sigma_f$), mating distance ($\sigma_m$), and competitive interaction distance ($\sigma_c$). Left plot: $\sigma_m = U(0.2, 2)$; $\sigma_f = 1$; $\sigma_c = 1$. Middle plot: $\sigma_m = 1$; $\sigma_f = U(0.2, 2)$; $\sigma_c = 1$. Right plot: $\sigma_m = 1$; $\sigma_f = 1$; $\sigma_c = U(0.2, 2)$.

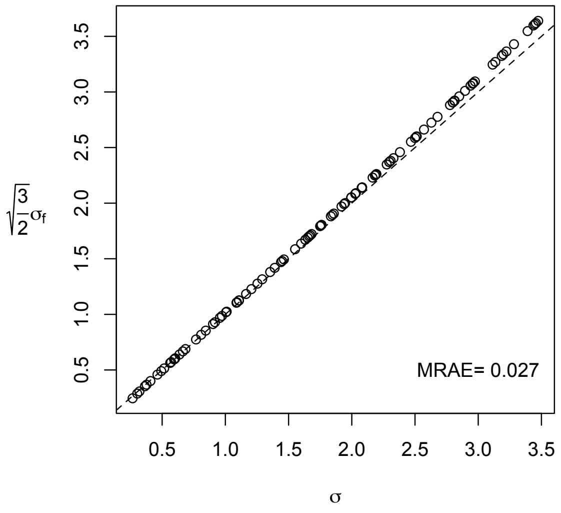


**Figure S2**. The x axis is true $\sigma$ measured during the simulation, whereas the y axis shows true $\sigma_f$ with the post hoc correction. In these simulations $\sigma_f = \sigma_m = \sigma_c$ (mother-offspring distance $\sigma_f$; mating distance $\sigma_m$; competitive interaction distance $\sigma_c$). MRAE is the mean relative absolute error.

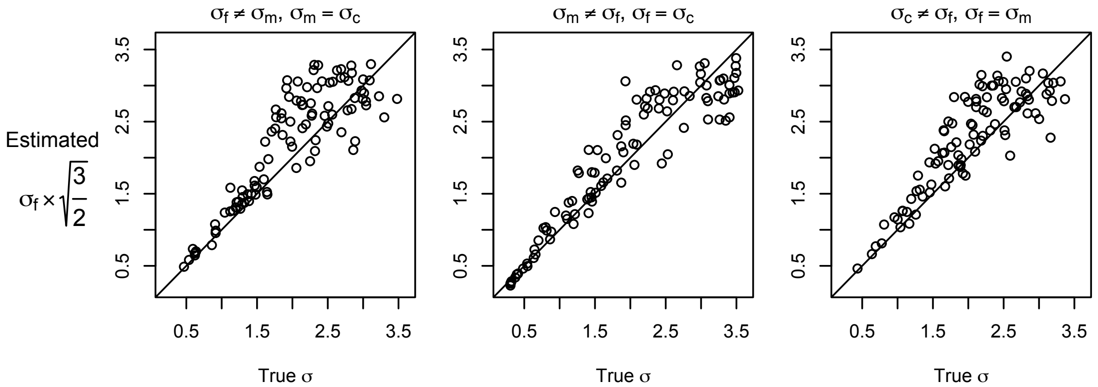


**Figure S3**. The x axis is true $\sigma$, and the y axis is estimated $\sigma_f$ multiplied by the post hoc correction, after training with $\sigma_f = \sigma_m = \sigma_c$ (mother-offspring distance $\sigma_f$; mating distance $\sigma_m$; competitive interaction distance $\sigma_c$). In the test data for each panel, either $\sigma_f$, $\sigma_m$, or $\sigma_c$ was varied independently (Uniform(0.2,3)) of the other two spatial parameters which were set to be equal (drawn from another Uniform(0.2,3)).

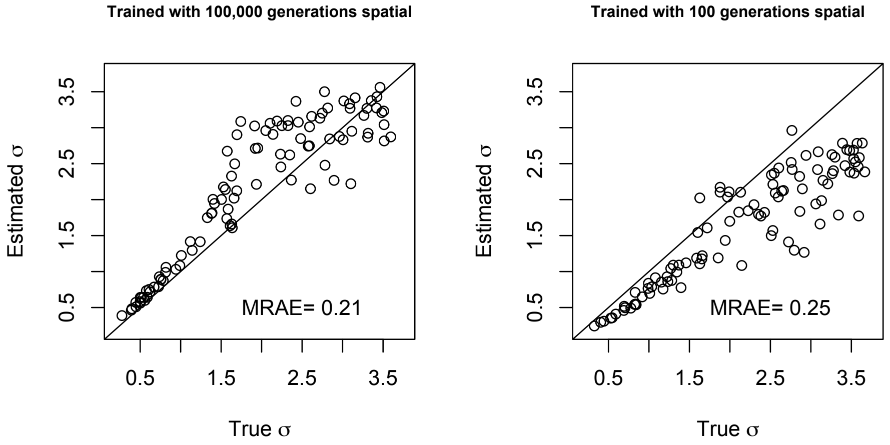


**Figure S4**. `disperseNN` trained with only 100,000 generations, or 100 generations, in spatial SLiM before recapitation with msprime (other parameters as in Parameter Set 2). Test data were full-spatial: SLiM was run until every tree had coalesced. MRAE is the mean relative absolute error.

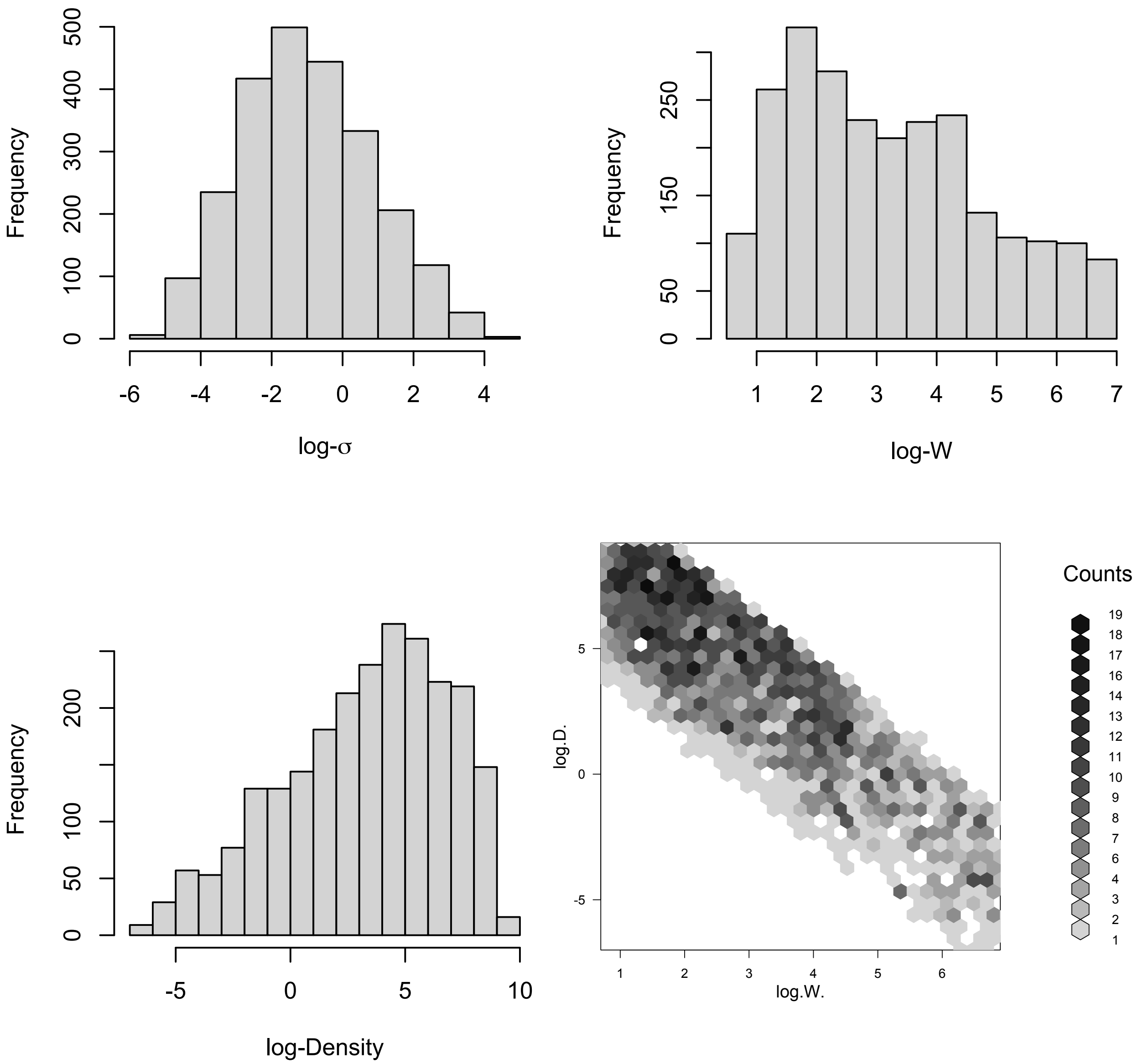


**Figure S5**. Realized training distributions for empirical analysis (Parameter Set 11). "D" is population density. "W" is habitat width. Some areas of parameter space could not be simulated due to population extinction or computational limitations.

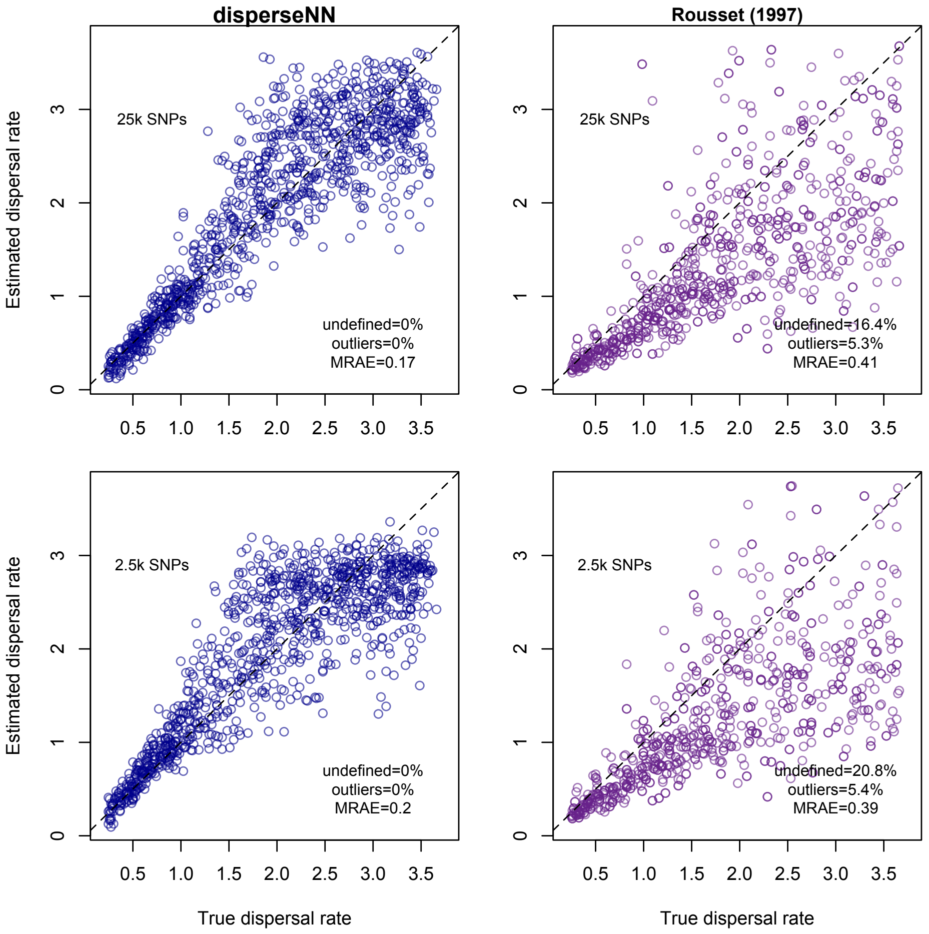


**Figure S6**. Comparison between `disperseNN` and Rousset's method using 10 individuals and varying SNP number (other parameters as in Parameter Set 1); compare with Figure 3. MRAE is the mean relative absolute error.

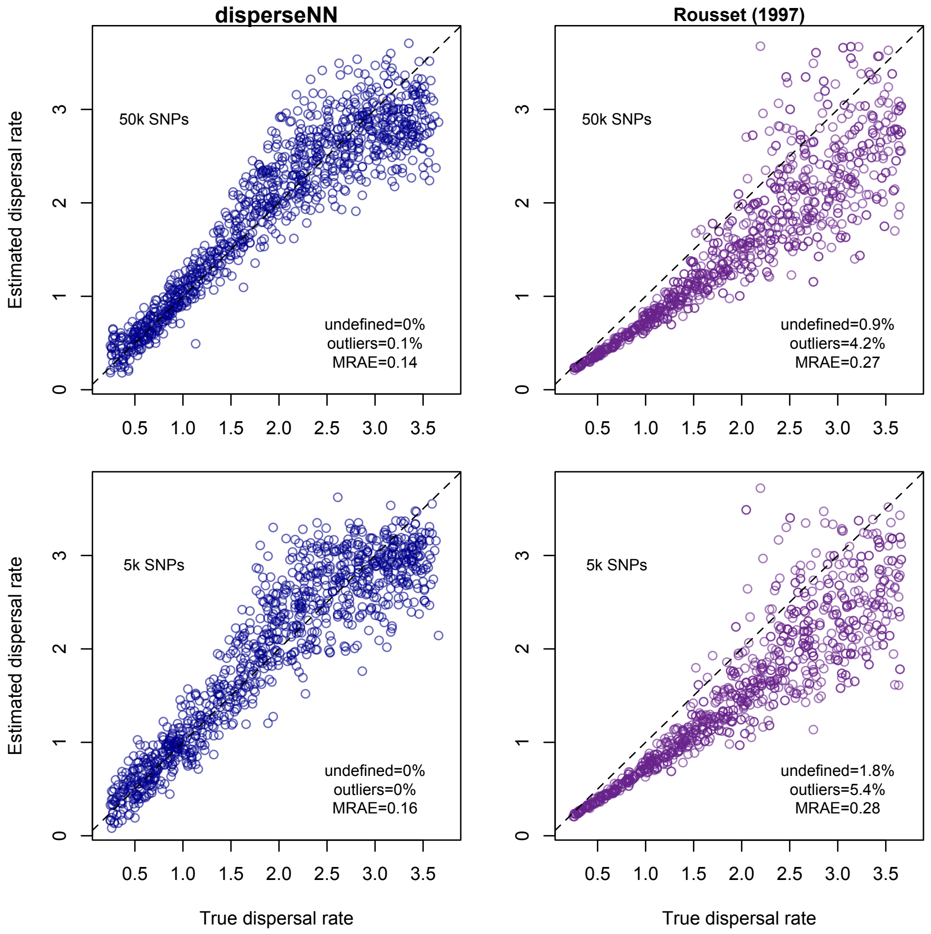


**Figure S7**. Comparison between `disperseNN` and Rousset's method using 100 individuals and varying SNP number (other parameters as in Parameter Set 1); compare with Figure 3. MRAE is the mean relative absolute error.

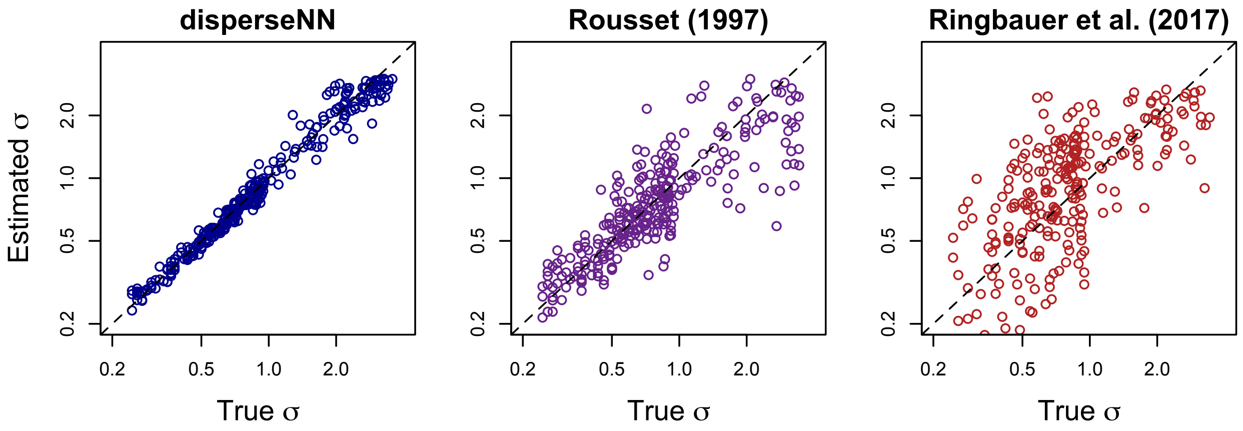


**Figure S8**. Predictions with log-transformation to show relative error ($n = 10$; Parameter Set 1). Data points in the larger half of the $\log(\sigma)$ range were down-sampled to one-half the number of points in the smaller half of the range to obtain roughly even density of points across the full range; before down-sampling, points were more dense towards the right-hand side which might give the false impression of larger variance.

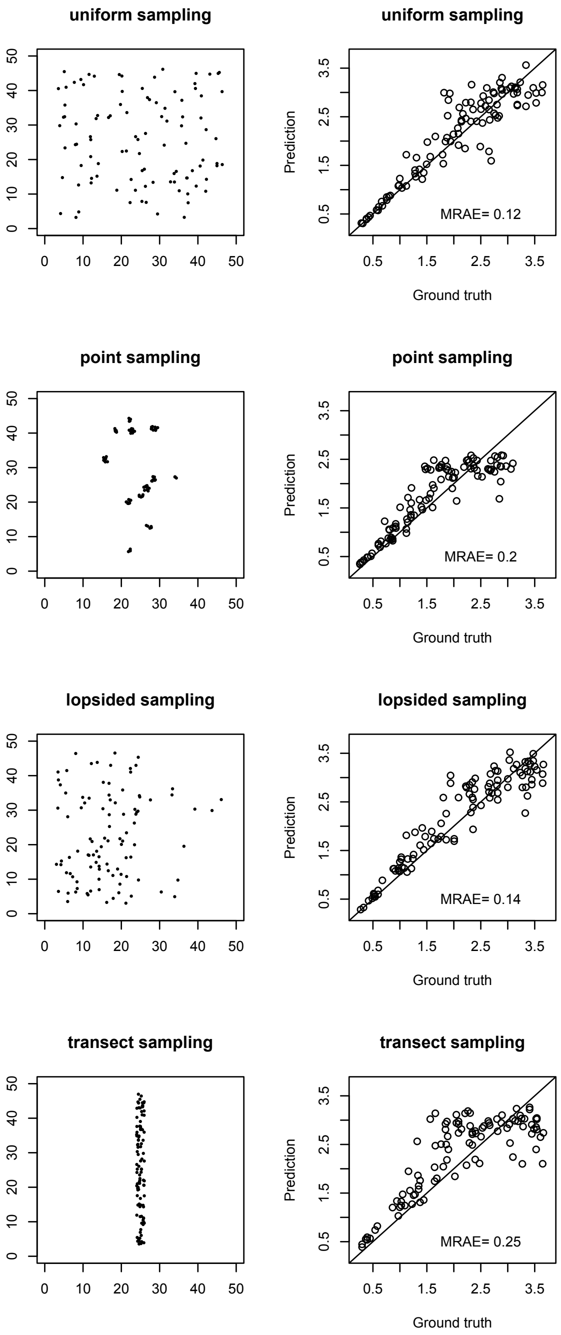


**Figure S9**. Exploring different sampling strategies. The left column shows the geographic distribution of sampling locations and the right column shows the `disperseNN` output, having trained with Parameter Set 2. For the 'point sampling' scenario, we used the sampling configuration from Munshi-South et al (2016); for this, we used the same subset of 12 sample locations used in our empirical analysis, except here we used more than one individual per sampling locality, ranging from $n = 4$ to $n = 11$ per location. MRAE is the mean relative absolute error.

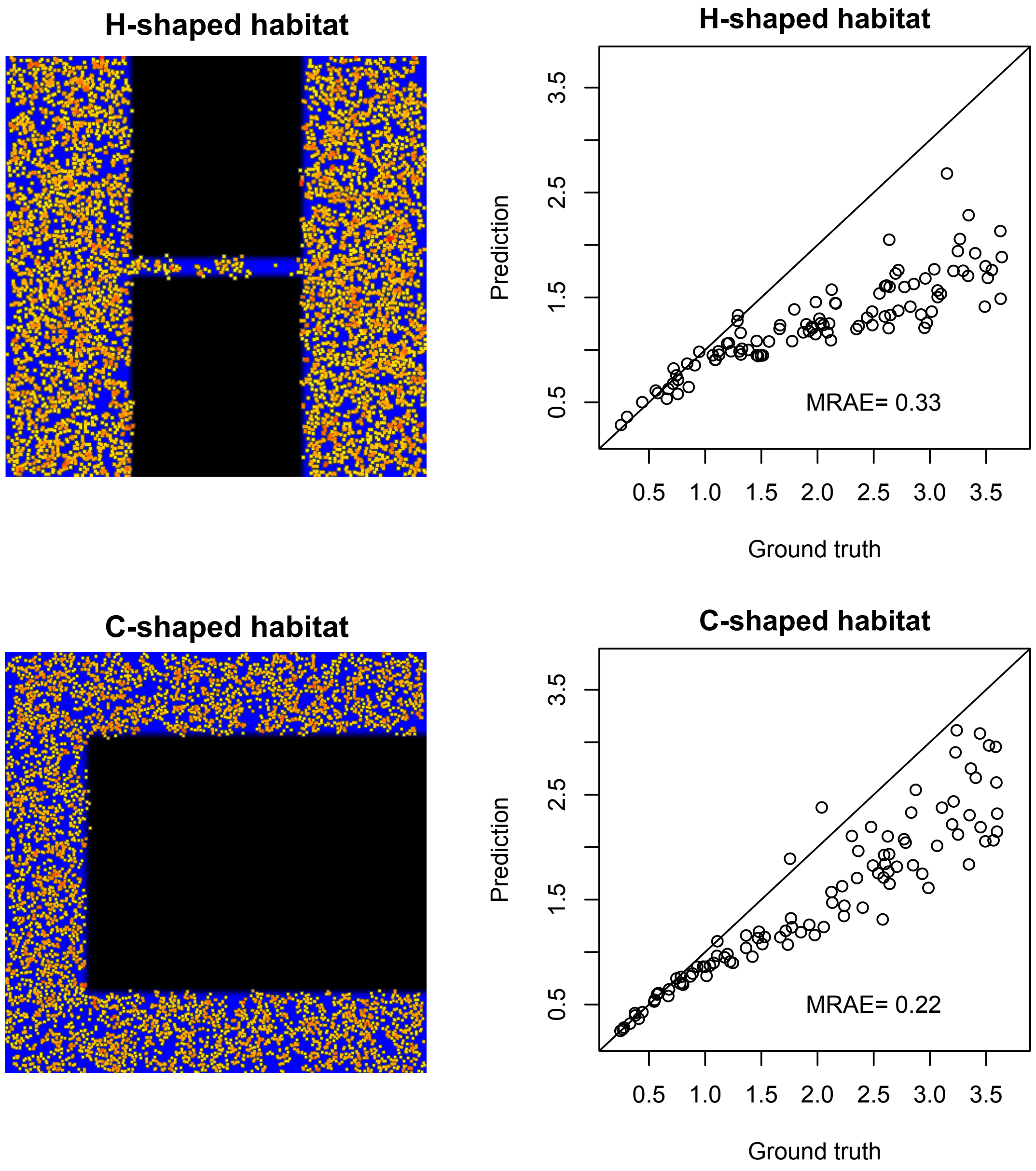


**Figure S10**. Exploring misspecified habitat shapes. The left column shows the simulation that produced the test data (screenshots of SLiM GUI): a square map (50 units wide) where individuals are yellow/orange, suitable habitat is blue, and unsuitable habitat is black. The right column shows the `disperseNN` output, having trained with a uniform (all blue) map (Parameter Set 2). The H-shaped habitat was inspired by McRae (2006) and the C-shaped habitat was inspired by McRae and Beier (2007). MRAE is the mean relative absolute error, and both axes are in units of $\sigma$.

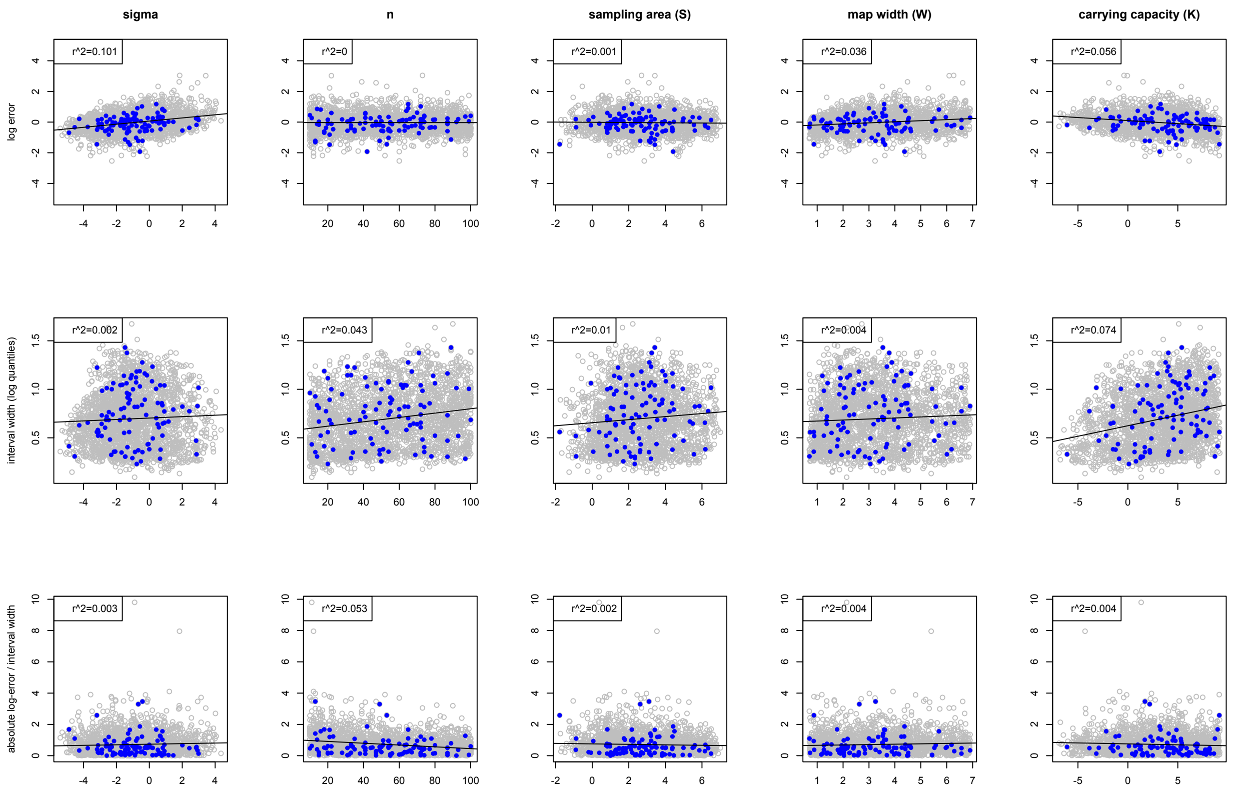


**Figure S11**. Exploring associations between five different predictor variables—(1) $\sigma$, (2) $n$, (3) sampling area, (4) map width, (5) carrying capacity—and three different response variables (A) log error, (B) the interval width of the log-transformed middle 95% range of the bootstrap distribution, and (C) absolute log-error divided by the interval width (Parameter Set 11). Shown are 2400 datasets including both held-out test data (blue; 100 datasets) and training data (grey; 2,300 datasets). The line of best fit and $r^2$ include all 2400 data points.

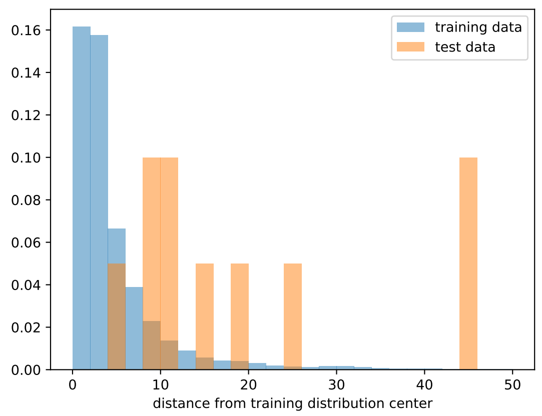


**Figure S12**. Mahalonobis distance from the center of the training distribution with respect to five summary statistics: nucleotide diversity, Tajima's D, inbreeding coefficient, observed heterozygosity, and expected heterozygosity (Parameter Set 11). "Test data" are the empirical datasets.

## References


Brad H McRae. Isolation by resistance. Evolution, 60(8):1551–1561, 2006.

Brad H McRae and Paul Beier. Circuit theory predicts gene flow in plant and animal populations. Proceedings of the National Academy of Sciences, 104(50):19885–19890, 2007.

Jason Munshi-South, Christine P Zolnik, and Stephen E Harris. Population genomics of the Anthropocene: Urbanization is negatively associated with genome-wide variation in white-footed mouse populations. Evolutionary applications, 9(4):546–564, 2016.
